# Low numbers of pre-leukemic fusion genes are frequently present in umbilical cord blood without affecting DNA damage response

**DOI:** 10.18632/oncotarget.16211

**Published:** 2017-03-15

**Authors:** Pavol Kosik, Milan Skorvaga, Matus Durdik, Lukas Jakl, Ekaterina Nikitina, Eva Markova, Katarina Kozics, Eva Horvathova, Igor Belyaev

**Affiliations:** ^1^ Cancer Research Institute, Biomedical Research Center, Slovak Academy of Sciences, Bratislava, Slovakia; ^2^ Cancer Research Institute, Siberian Branch of the Russian Academy of Medical Sciences, Tomsk, Russia

**Keywords:** pre-leukemic fusion genes, stem cells, DNA damage response, apoptosis

## Abstract

Despite widely accepted notion that many childhood leukemias are likely developed from hematopoietic stem/progenitor cells (HSPC) with pre-leukemic fusion genes (PFG) formed in embryonic/fetal development, the data on PFG incidence in newborns are contradictive. To provide a better understanding of a prenatal origin of leukemia, umbilical cord blood from 500 newborns was screened for the presence of the most frequent PFG associated with pediatric B-cell acute lymphoblastic leukemia. This screening revealed relatively high incidence of ETV6-RUNX1, BCR-ABL1 (p190) and MLL-AF4 at very low frequencies, averaging ~14 copies per 100,000 cells. We assume that most of these PFG might originate relatively late in embryonic/fetal development and will be eliminated later during postnatal development. The obtained results suggested that higher PFG copy numbers originating in specific time windows of the hematopoietic stem cell hierarchy may define a better prognostic tool for the assessment of leukemogenic potential. We have observed no significant effect of low-copy PFG on radiation-induced DNA damage response, accumulation of endogenous DNA double-stranded breaks, and apoptosis in either lymphocytes or HSPC. Imaging flow cytometry showed lower level of γH2AX foci in HSPC in comparison to lymphocytes suggesting better protection of HSPC from DNA damage.

## INTRODUCTION

Acute lymphoblastic leukemia (ALL) is the most common malignancy in childhood. ALL arises from clonal proliferation of hematopoietic stem cell/progenitor cell (HSPC), replacing normal hematopoietic cells in the bone marrow (BM) [1, 2]. The development of pediatric ALL is a multistep process driven by the accumulation of two types of genetic abnormalities, (i) 1^st^ hit, often a chromosomal translocation generating a pre-leukemic fusion gene (PFG), and (ii) 2^nd^ hit, often point mutations, deletions, duplications. Several data, including studies in identical twins and with neonatal spots (Guthrie cards) have suggested that the initiation of childhood ALL-associated chromosomal translocations may arise *in utero* during fetal hematopoiesis [3–9]. The resultant fusion gene product with a novel activity, different from the activities of individual partner genes, produces a persistent, but covert pre-leukemic clone [10–12]. In their pioneer study, the Greaves’ group reported ~1.06% incidence of TEL-AML1/ETV6-RUNX1, the most common ALL-associated PFG, in umbilical cord blood (UCB) samples from England and Italy [10]. These data were further confirmed by other groups by screening UCB of newborns from Czech Republic, Japan, USA, and Slovakia [10, 13, 14] but challenged by the Danish group reporting that the incidence of ETV6-RUNX1 is the same as the incidence of ETV6-RUNX1 related ALL [15]. The purported coincidence of overt ALL and related PFG suggested an effective approach for early ALL diagnostics by screening UCB of newborns. Thus, validation of the PFG incidence in UCB become of critical importance for leukemia risk estimation and diagnostics.

All cells are equipped with DNA damage response (DDR) pathways that trigger DNA repair, cell cycle arrest, and, if needed, apoptosis, to eliminate DNA damage or damaged cells [16]. Apoptosis (controlled cell death) is one of the two mechanisms by which cell death occurs (the other being the pathological process of necrosis). It is responsible for the physiological elimination of cells and appears to be genetically programmed. DNA damage and apoptosis are key factors in the accumulation of changes associated with cell transformation. PFG are a consequence of chromosomal translocations which are assumed to be initiated by DNA double strand breaks (DSB), which occur as a result of endogenous insults, such as attack by oxygen radical species produced during metabolism, or from exogenous insults, such as ionizing radiation [17]. DSB and their repair have been implicated in the generation of key chromosomal translocations in both childhood and adult acute myeloid and lymphoid leukemias [18]. Therefore, the combination of increased DSB and impaired repair could create the environment for the acquisition of genetic alterations, which is a hallmark of leukemogenesis. Recent studies suggest that BCR-ABL1 and ETV6-RUNX1 may directly lead to genetic instability through induction of reactive oxygen species (ROS) and the accumulation of DNA damage including DSB [19–21]. This observation also suggests that increased ROS production induced by PFG products could be a common mechanism driving leukemogenesis.

Several methods have been developed for a qualitative or quantitative measurement of DSB formation in cells, including analysis of DNA repair foci and comet assay. The former method is based on microscopic visualization of discrete foci called DNA repair foci [22–25], which are formed at the site of DSB as a result of the recruitment of DDR proteins. A key event in DNA repair focus formation is the rapid phosphorylation of histone H2AX (γH2AX) that provides a chromatin scaffold formed on a 2-Mb sized chromatin domain containing DSB and functions by recruiting proteins involved in DSB repair [26]. DNA repair foci are dynamic structures containing thousands of copies of proteins involved in various aspects of DSB repair and signaling, including p53-binding protein 1 (53BP1) that is able to migrate across a nucleus and bind to DNA repair foci within seconds of DSB induction [27]. The comet assay is a sensitive and rapid method for detecting DNA strand breaks in individual cells [28].

In our recent study, we screened UCB samples from 200 Slovak newborns for the presence of three most common pediatric B-ALL associated PFG: ETV6-RUNX1, BCR-ABL1, and MLL-AF4 [14]. The present work: (i) enlarged the studied group by the addition of 300 UCB samples, (ii) tested and validated the PFG positivity by replicated RT-qPCR and cDNA sequencing. Using complementary techniques, endogenous DSB, apoptosis/necrosis (in lymphocytes/HSPC), and radiation-induced DDR in MNC from UCB of PFG-positive (PFG^+^) and matched PFG-negative (PFG^−^) probands were studied and compared.

## RESULTS

### Incidence of PFG associated with childhood B-cell ALL in UCB of newborns

In our previous study, 200 UCB probands were screened for the presence of the most common PFG associated with childhood B-cell ALL using the RT-qPCR method with a sensitivity of approximately 1–3 copies per 100,000 cells [14]. About 1/4 of 20 positive samples were randomly selected for validation by a reference laboratory in the National Institute of Oncology, Bratislava, Slovakia. Even after applying this validation rate, we achieved relatively high incidence of studied PFG, namely ~6.25% for BCR-ABL1 (p190), ~4% for ETV6-RUNX1, and ~0.75% for MLL-AF4. To improve the accuracy of PFG screening, we added (i) another 300 UCB probands to the previously screened sample set, and (ii) a validation step consisting of replicated RT-qPCR verification runs and sequencing in an attempt to reduce false signal positivity which might be considered as one of the causes of unexpectedly high PFG frequencies that we and others have recorded [10, 14].

**Figure 1:**
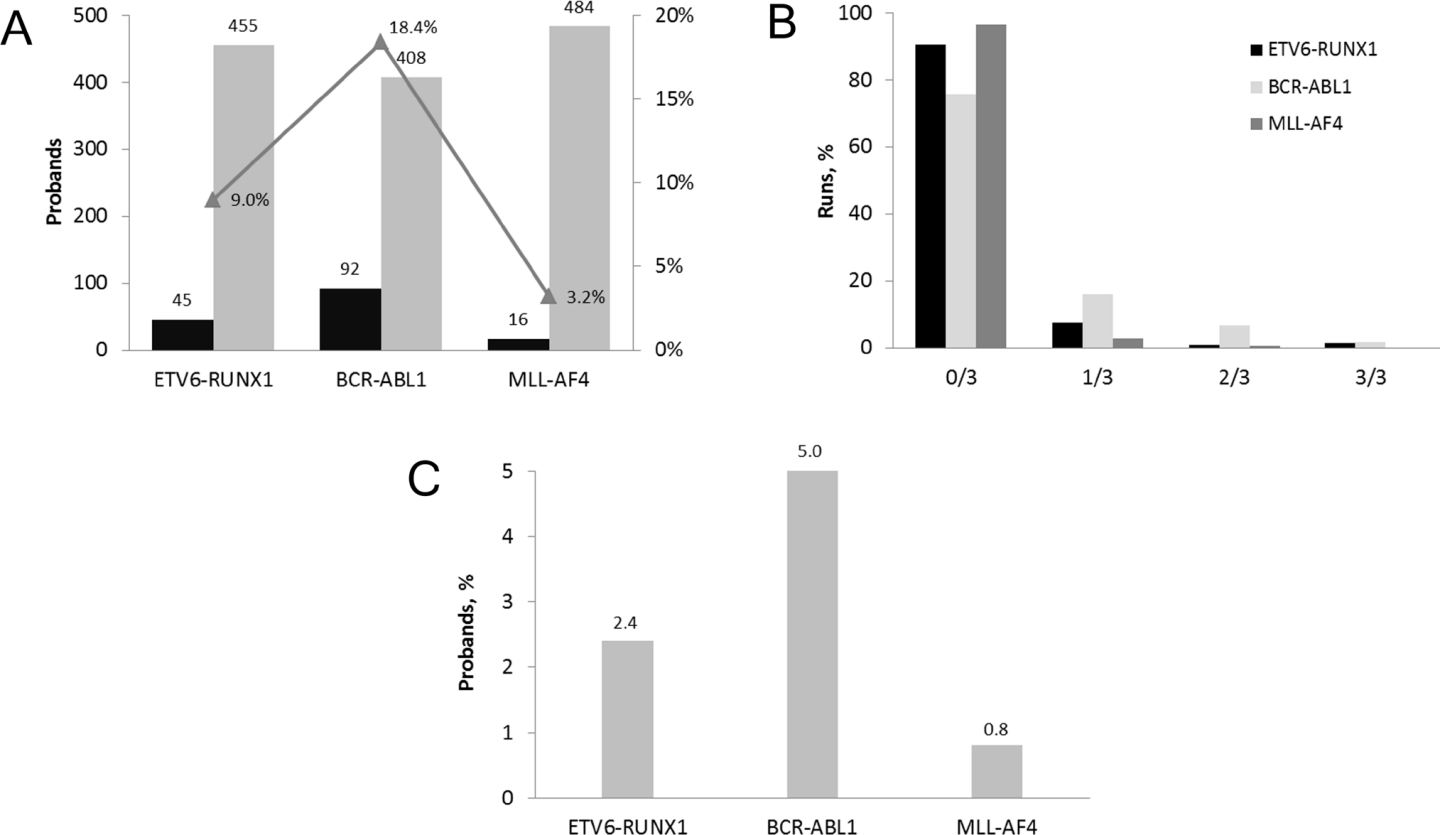
Incidence of PFG associated with childhood B-cell ALL in UCB of Slovak newborns (**A**) The number of probands tested for PFG in 500 primary RT-qPCR runs. The incidence of PFG positive probands is displayed in percentage. (**B**) Distribution of PFG positive samples among 500 UCB probands tested by RT-qPCR in 1654 runs. The percentage of negative, 0/3, and positive i.e. 1/3, 2/3, 3/3 runs each performed in triplicate is shown for each type of PFG. (**C**) Incidence of PFG positive probands after applying the total validation rate, consisting of 30% refined RT-qPCR validation rate and 90.9% validation rate estimated by sequencing.

From the total number of 500 UCB probands, 133 probands were tested positive, i.e. PFG was found at least in one of triplicate samples by the primary RT-qPCR. 92 probands were positive for BCR-ABL1, 45 for ETV6-RUNX1, and 16 for MLL-AF4 (Figure 1A). In 20 probands, we detected two different PFG and one proband was tested positive for all three PFG. Cycle threshold (C_t_) of PFG^+^ samples averaged 37 with a number of PFG copies averaging 14 per 100,000 cells. The median represented three PFG copies per 100,000 cells. Even though a small number of samples contained multiple PFG copies, this was usually at less than 6 copies per 100,000 cells (Supplementary Figure 1). The C_t_ value and the number of ABL1 control gene copies averaged 26 and 19482, respectively, conforming reliable application of the RT-qPCR method.

As shown in our previous report, validation of the PFG^+^ samples in a reference laboratory revealed that 5 of 20 PFG^+^ samples were confirmed with the same screening method, setting the validation rate at 25% [14]. In this study, we refined the RT-qPCR validation rate with a larger group of probands. This validation was performed with the same screening method starting from a new frozen MNC aliquot for each proband. Samples from ninety PFG^+^ probands were repeatedly analyzed altogether in 229 RT-qPCR runs (Supplementary Figure 2). The validation rate from two, three and four repeated RT-qPCR runs was estimated to be 27/90 (30%). The refined RT-qPCR validation rate was comparable to the 25% validation rate obtained previously in the reference laboratory [14].

Quantitative analysis of all data has confirmed the previously observed trend of very low PFG levels in positive UCB samples manifesting as (i) 1/3 positivity of triplicate measurements in the majority of samples (75.73, 90.54, and 96.6% for BCR-ABL1, ETV6-RUNX1, and MLL-AF4, respectively), and (ii) very low numbers of PFG copies in positive samples (Figure 1B).

Twenty-two samples, from which one was tested positive in one run, 17 in two runs, and 4 in three RT-qPCR runs, were validated by sequencing (Table 1). Twenty of them were found to contain the expected PFG sequence (i.e. sequence comprising the site of fusion gene), thus setting the sequencing validation rate to 20/22 (90.9%) (Supplementary Data 1). The remaining two probands could not be confirmed by sequencing due to the absence of PFG-specific sequences.

**Table 1:** UCB samples defined PFG positive by RT-qPCR in replicated runs, each in triplicate, and subjected to sequencing

# Proband	PFG	RUN-1	RUN-2	RUN-3	RUN-4	TOTAL RUNS	RESULT
P 138	BCR-ABL	0/3	2/3	2/3		3	Positive
P 141	BCR-ABL	1/3	0/3	3/3	1/3	4	Positive
**P 143**	**ETV6-RUNX1**	**3/3**	**3/3**	**3/3**		**3**	**Positive**
P 144	BCR-ABL1	3/3	2/3	0/3		3	Positive
**P 145**	**BCR-ABL1**	**2/3**	**2/3**	**0/3**	**1/3**	**4**	**Positive**
P 146	MLL-AF4	1/3	2/3	0/3		3	Positive
P 203	BCR-ABL1	2/3	0/3	1/3		3	Positive
P 206	BCR-ABL1	0/3	1/3	1/3	2/3	4	Positive
P 215	BCR-ABL1	3/3	0/3	3/3		3	Positive
P 216	ETV6-RUNX1	1/3	1/3	0/3		3	Positive
P 217	BCR-ABL1	2/3	2/3	0/3		3	Positive
**P 218**	**ETV6-RUNX1**	**1/3**	**1/3**			**2**	**Positive**
**P 219**	**ETV6-RUNX1**	**1/3**	**0/3**	**2/3**		**3**	**Positive**
**P 230**	**ETV6-RUNX1**	**1/3**	**2/3**			**2**	**Positive**
**P 233**	**BCR-ABL1**	**1/3**	**2/3**			**2**	**Positive**
**P 239**	**BCR-ABL1**	**1/3**	**1/3**			**2**	**Positive**
**P 310**	**BCR-ABL1**	**1/3**	**0/3**	**1/3**		**3**	**Positive**
P 377	BCR-ABL1	2/3	1/3			2	Positive
**P 522**	**BCR-ABL1**	**1/3**	**1/3**			**2**	**Positive**
**P 546**	**BCR-ABL1**	**1/3**				**1**	**Positive**
P 137	MLL-AF4	1/3	2/3			2	Negative
P 292	MLL-AF4	1/3	1/3			2	Negative

Samples selected for subsequent analysis of endogenous DSB and DNA damage response are in bold.

Figure 1C summarizes the RT-qPCR data from 500 UCB probands obtained after applying the validation rates, i.e. the 30% refined RT-qPCR validation rate and the 90.9% sequencing validation rate. Finally, the incidence of the B-ALL associated PFG as measured in 500 UCB probands was estimated as follows: BCR-ABL1 at 5%, ETV6-RUNX1 at 2.4%, and MLL-AF4 at 0.8% (Figure 1C).

### Endogenous DSB, apoptosis/necrosis, and radiation induced DNA damage response

Ten PFG^+^ UCB samples, in which particular PFG were validated by both RT-qPCR and sequencing (Table 1), were further studied for (i) endogenous DSB and apoptosis/necrosis monitored by γH2AX/53BP1 DNA repair foci and flow cytometry, respectively, (ii) radiation-induced DNA damage assessed by comet assay. Ten matched UCB samples without PFG were chosen for comparison (Supplementary Table 1).

**Figure 2:**
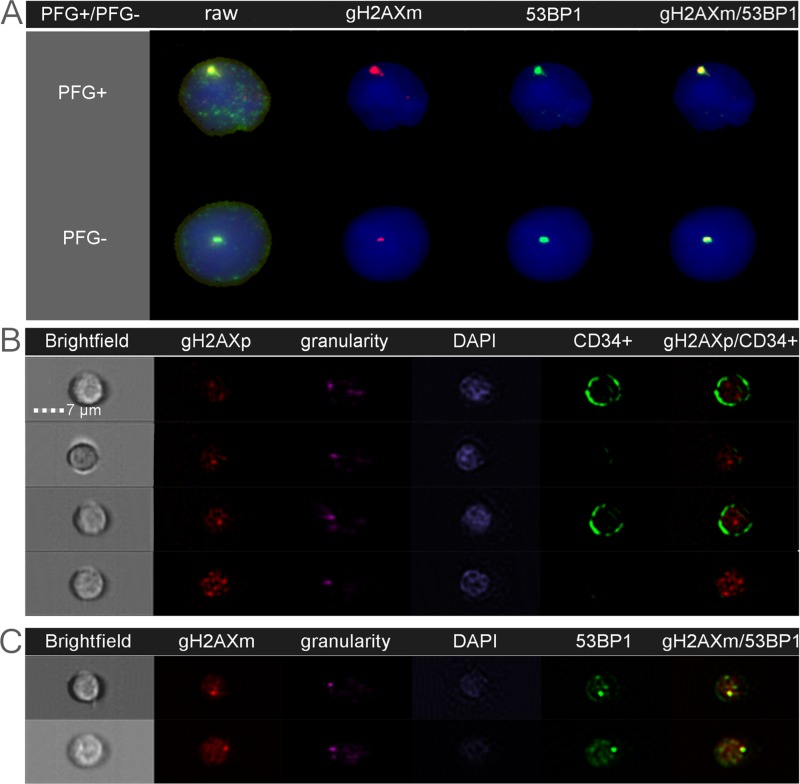
Endogenous DSB analyzed as DNA repair foci by fluorescent microscopy (**A**) and imaging flow cytometry (**B**–**C**). Nuclear DNA is stained in blue/purple by DAPI. The gH2AX and 53BP1 foci localized at endogenous DSB are shown in red and green, respectively. Co-localization of γH2AXm and 53BP1 is seen in yellow. (A): The figure shows representative images of lymphocytes from the BCR-ABL1 positive (PFG^+^) and PFG-negative (PFG^−^) probands. A similar level of γH2AXm and 53BP1 foci was observed in both probands. (B): Representative images of four MNC analyzed for γH2AXp. Bright field and granularity was used to detect morphological properties of cells. From those, two cells are lymphocytes and other two cells are HSPC containing CD34 marker (green signal) on their membranes. (C): Two representative MNC, which were simultaneously analyzed for γH2AXm and 53BP1 foci.

**Figure 3:**
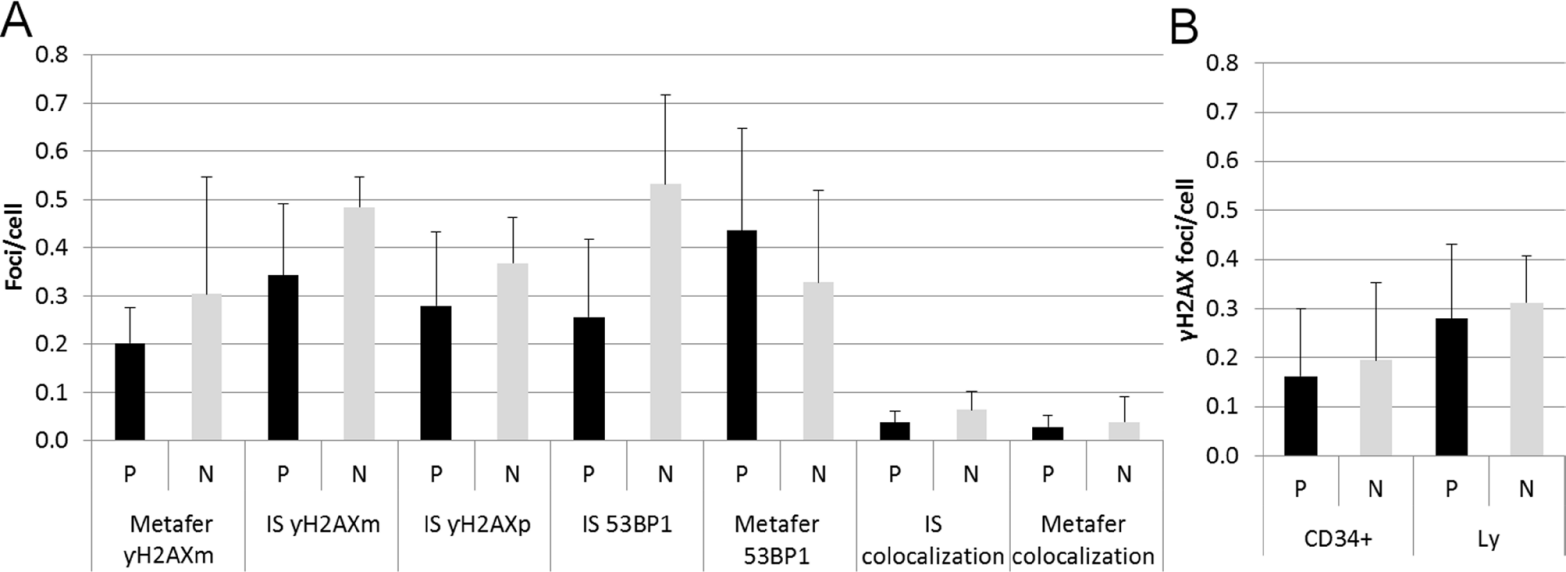
Endogenous DSB (**A**) The graph shows summary data for γH2AXm, γH2AXp, 53BP1 foci, and 53BP1/γH2AX co-localization obtained by the ImageStream (IS) and Metafer systems in lymphocytes from PFG positive (P) and PFG negative (N) probands. (**B**) The bar chart indicates the level of γH2AXp foci in HSPC (CD34^+^) and lymphocytes (Ly) from UCB of PFG-positive (P) and matched PFG-negative (N) probands detected by imaging flow cytometry using the ImageStream system. In both graphs, mean ± SD from 10 probands is shown for each data point.

Since HSPC are considered to be the main target for the origin of leukemia and each PFG requires DSB for its formation, we performed a systematic analysis of endogenous DSB in HSPC and differentiated lymphocytes using generally accepted DSB molecular markers, γH2AX and 53BP1. Two independent approaches for measuring the level of endogenous DNA repair foci, namely fluorescence microscopy by Metafer system (Figure 2A) and imaging flow cytometry by ImageStream system (Figure 2B, 2C) were used and compared. In line with previously published data, some γH2AX and 53BP1 foci did not co-localize due to various kinetics of γH2AX and 53BP1 at the locations of DSB [29]. By analyzing γH2AX, 53BP1 foci and their co-localization using imaging flow cytometry we did not find significant difference in endogenous DSB between ten PFG^+^ and ten PFG^−^ samples (*p* = 0.16, ANOVA) (Figure 3A). Analysis by fluorescent microscopy using Metafer system confirmed the data obtained by imaging flow cytometry and did not reveal any statistically significant difference in endogenous DSB between PFG^+^ and PFG^−^ samples (*p* = 0.22, ANOVA) (Figure 3A). Similar values of endogenous DSB per cell (~0.2–0.6) were obtained within all groups (CD34^+^, Ly, PFG^+^, PFG^−^) as confirmed using various antibodies (γH2AXp, γH2AXm, 53BP1). However, enumeration of γH2AXp by imaging flow cytometry as shown in Figure 3B indicated a slightly lower level of endogenous DSB in CD34^+^ HSPC than in lymphocytes (*p* = 0.0127, two tailed *t*-test) (Figure 3B) suggesting better protection of HSPC from endogenous DNA damage. As a positive control, we analyzed induction of γH2AX/53BP1 foci 30 min post-irradiation with γ-rays in the dose range of 2–50 cGy. We found statistically significant increase of γH2AX and 53BP1 foci already at 2 cGy (data not shown) confirming very high sensitivity of our assays [29].

**Figure 4:**
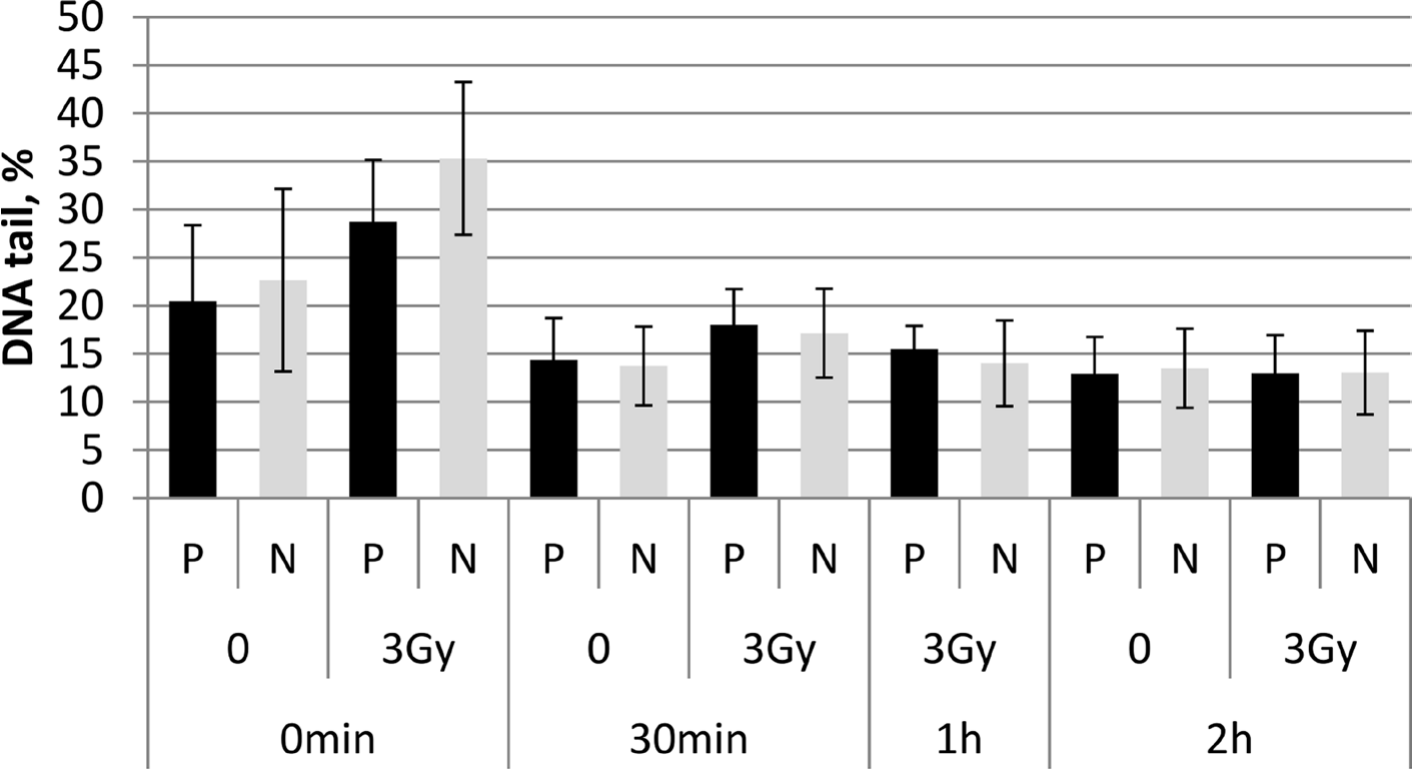
Alkaline comet assay The bar chart shows summary data obtained with the alkaline comet assay at 0 h, 30 min, 1 h, and 2 h after irradiation of UCB MNC from PFG positive (P) and PFG negative (N) probands with 3 Gy. Mean ± SD of three independent experiments is shown for each data point.

To investigate the possible effect of PFG on DDR, radiation-induced DNA breaks and their repair was monitored in UCB mononuclear cells (MNC) from PFG^+^ and PFG^−^ samples. The alkaline comet assay was applied, which was designed for all kinds of DNA breaks. Irradiation with 3 Gy of γ-rays significantly increased DNA in the comet tails immediately after irradiation in both PFG^+^ and PFG^−^ samples (*p* = 0.026 and 0.007, respectively, two tailed *t*-test). However, no difference in DNA damage or in kinetics of DNA repair was detected when PFG^+^ and PFG^−^ samples were compared (Figure 4).

**Figure 5:**
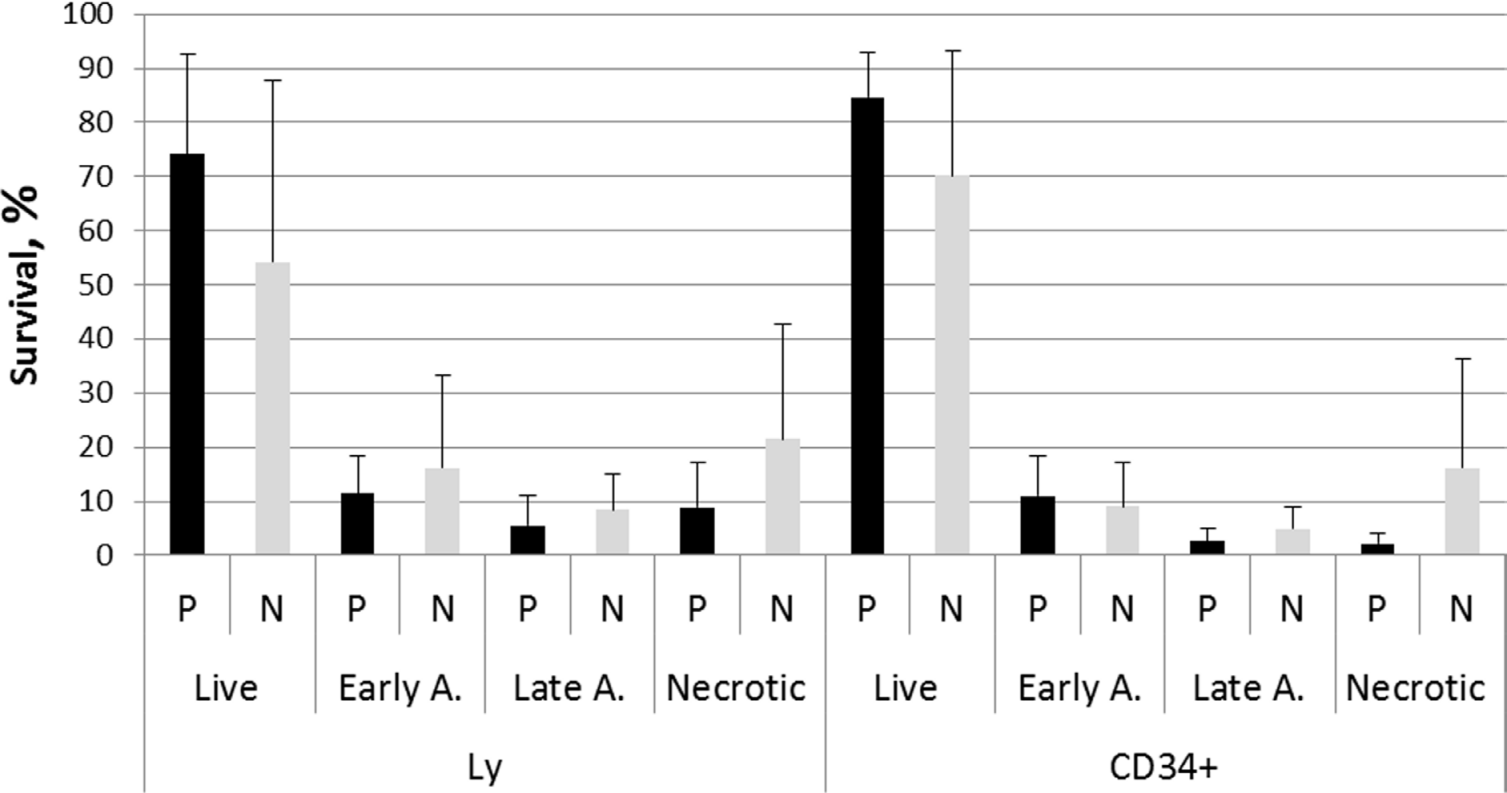
Survival ability of UCB cells from ten PFG positive samples (P) verified by sequencing and ten matched PFG negative controls (N) Graph shows live cells, early apoptotic, late apoptotic, and necrotic cells in lymphocytes (Ly) and HSPC (CD34^+^) measured after thawing the MNC and 30 min incubation. Mean ± SD of UCB cells from ten probands expressed in % is shown for each data point.

Cell viability was monitored in unirradiated lymphocytes and HSPC from matched PFG^+^ and PFG^−^ samples by measuring live, early apoptotic, late apoptotic and necrotic cells using fluorescence-activated cell sorting (FACS) analysis. CD34^+^ cell incidence in UCB MNC averaged 1.48% and 1.43% in PFG^−^ and PFG^+^ samples, respectively. Obtained data did not show any significant difference in cell viability between PFG positive and negative groups (*p* = 0.12, ANOVA). While it was a trend in higher survival of CD34^+^ than CD34^−^ cells, no statistically significant difference was found between these cell populations (*p* = 0.055, ANOVA) (Figure 5). We concluded that UCB cells from both PFG^+^ and PFG^−^ probands exhibited similar endogenous DNA damage, apoptosis and radiation-induced DNA damage response.

## DISCUSSION

In this study, mononuclear cells of 300 UCB from the Slovak National Birth Cohort were screened for the presence of most common and prognostically important PFG of B-lineage ALL, i.e. ETV6-RUNX1, MLL-AF4 and BCR-ABL1 (p190). Ultimately, in combination with our previously published data [14] the cohort of 500 newborns was screened. The quantitative analysis of summary data confirmed the tendency observed in the first 200 samples, namely an extremely low level of studied PFG in few positive samples, reaching usually < 6 copies per 100,000 cells. Our data clearly show that the amount of PFG in the majority of PFG^+^ UCB samples is very close to the threshold of RT-qPCR sensitivity, which was reached at approximately 1–3 copies per 100,000 cells in our hands [14]. This fact stresses the sensitivity limitations of the screening method and suggests that only an approximate estimation of PFG incidence can be made if the obtained data are only slightly above the sensitivity level. Our data also indicate that the development and application of new methodological approaches ensuring sensitivity at least 1 × 10^−6^ are highly encouraged for further research.

Along with refined validation of PFG positivity in replicated RT-qPCR runs we introduced another validation step, namely sequencing the selected PFG^+^ RT- qPCR products. The sequencing validation rate of 90.9% revealed relatively low level of false positives. By applying the refined RT-qPCR and sequencing validation rates, the overall estimated incidences of PFG in Slovak population made on UCB from five hundred newborns are as follows: ~5% for BCR-ABL1 (p190), ~2.4% for ETV6-RUNX1, and ~0.8% for MLL-AF4. Of note, the refined incidence of ETV6-RUNX1, the most common ALL-associated PFG in UCB, is similar to the data by Mori et al. who identified ETV6-RUNX1 fusion in 6 of 567 (~1.06%) UCB samples from England and Italy [10]. Importantly, the presence of corresponding chromosomal translocation in positive samples was confirmed by FISH analysis [30]. In line with these data, Eguchi-Ishimae et al. examined 67 cord blood samples for the existence of in-frame ETV6-RUNX1 fusion transcripts and one cord blood sample (1.5%) was positive as detected by nested RT-PCR [31]. The study by Zuna et al. revealed the presence of ETV6-RUNX1 in 5 of 253 (~1.98%) cord blood cells of healthy newborns from the Czech Republic [13]. However, contrary to our results, the MLL-AF4 and BCR-ABL1 (p190) in this study were not detected in UCB of 103 newborns. It should be noted that Zuna et al. used different techniques for screening ETV6-RUNX1 and BCR-ABL1/MLL-AF4, RT-qPCR and RT-PCR, respectively. While the ETV6-RUNX1 data were obtained by the same RT-qPCR method as in present study, the sensitivity of RT-PCR was lower, ~1 × 10^−4^ [32]. Thus, the data on BCR-ABL1/MLL-AF4 are not directly comparable between our and Zuna's studies. More recently, Ornelles et al. reported the presence of ETV6-RUNX1 fusion transcript in 5 of 210 (~2.4%) in UCB samples from the United States using a nested PCR assay [33]. Overall, the ~2.4% incidence of ETV6-RUNX1 in UCB of Slovak newborns, come close to aforementioned data in the range of 1 to 2.4%. Accordingly, it is believed that the pre-leukemic state is not rare, with greater than 1% of individuals having recognized pre-leukemic lesions [34].

Our data together with above mentioned reports support Model A suggesting that the initiating genetic event, i.e. ETV6-RUNX1 is ~100-fold more common than the overt disease [35]. This is in contrary to Model B which postulates that up to 100% of ETV6-RUNX1 carriers will develop ETV6-RUNX1^+^ ALL [15]. Of note, the Model B is based on the data by Danish group [15], which reported about 100-fold lower incidence of ETV6-RUNX1 as compared to other studies [10, 13, 31, 33] and present investigation.

It follows from our data that screening UCB MNC for the presence of PFG cannot give the definite answer to the question of whether the identified fusion gene will increase the risk of leukemia in its carriers during their postnatal development. Our data also provide evidence that the UCB MNC screening should be substituted for a different approach which allows for reducing the number of cells to be screened by sorting the cells with a leukemogenic potential–this cell population will also include pre-leukemic cells containing an initiating genetic lesion, e.g. PFG. In this way, the majority of cells, i.e. cells with no self-renewal capacity, which cannot cause leukemia but still may be positive for a leukemia-associated PFG, will be excluded. Within the population of cells capable of self-renewal and differentiation, cells containing PFG can be identified, separated and characterized in detail based on their membrane-specific antigens, their position within hematopoietic cell hierarchy, and their self-renewal and differentiation capacity. Identification of the PFG in the UCB/BM cells with leukemogenic potential may indicate an increased risk leukemia development in its carrier. Therefore, a future challenge is PFG screening in cells with leukemogenic potential. Isolation and a detailed characterization of these pre-leukemic cells may be important for the leukemia risk assessment as well as for donor-cell derived leukemia (DCL) as it might be caused by the covert pre-leukemic HSPC present in donor cord blood or bone marrow. The available data show that although the incidence of DCL is very low, ranging from 0.16% up to 5%, its median survival, however, is extremely low, reaching only five and six months using UCB and BM for transplantation, respectively [36, 37]. Importantly, we established that the frequency of PFG expression in neonatal cord blood samples is different for different fusion genes of BCR-ABL1 (p190), ETV6-RUNX1, and MLL-AF4. The formation of different PFG within different *in utero* developmental stages may underlie the difference in their incidence established in this study. Thus, the target cell may be different for the different fusion genes. Proximity patterns of chromosome territories may define the subpopulation of cells where specific PFG arise as result of chromosomal exchanges between proximally contacting genes [38]. In particular, BCR and ABL1 loci were found to be in mutual proximity in UCB HSPC [39]. This might contribute to a relatively higher frequency of BCR-ABL1 (p190) in UCB as compared to ETV6-RUNX1.

Undoubtedly, the induction of DSB is a key event in the formation of all PFG. The cells in newborns predisposed to leukemia may hypothetically have impaired DDR and thus increased level of endogenous DSB if the individual's genotype predisposes cells to DSB differences. According to this hypothesis, the cells in PFG^+^ newborns predisposed to leukemia may imminently have impaired DDR and increased level of endogenous DSB regardless type of PFG and stage of development. If the hypothesis on increased DSB in PFG^+^ UCB cells would be confirmed it might result in a cost effective clinical tool for leukemia risk estimation. Thus, we aimed to compare DDR between PFG positive and negative UCB. To this aim, DDR in ten PFG^+^ UCB MNC samples, which were validated by sequencing, was analyzed in comparison with ten matched PFG^−^ controls. We used the highly sensitive γH2AX/53BP1 foci approach to quantify spontaneous DSB in human HSPC and lymphocytes. Our data did not show any difference between PFG positive and negative groups, neither in HSPC nor in lymphocytes, suggesting that DDR is not impaired in MNC of the PFG^+^ probands. These γH2AX/53BP1 data was further confirmed by the measurements of apoptosis and DNA damage and repair using the comet assay.

Hypothetically, cord blood negative for the three fusion genes screened could be positive for other fusions or other leukemia/cancer-associated genetic changes. However, we have analyzed most frequent PFG related to ALL. Thus, a chance that cord blood may contain other ALL related PFG is relatively low. In total, we have screened almost hundred UCB for DSB (unpublished data). Our data for DSB in the PFG positive UCB cells fit to the very low range of variability in these samples supporting that tested PFG do not correlate with increased DSB.

To conclude, our data show that the low number of PFG, on average ~14 copies per 100,000 MNC does not correlate with impaired DDR neither in HSPC nor in lymphocytes. However, our data do not exclude that a minor fraction of UCB leukemogenic stem cells harboring PFG formed within specific time windows of prenatal development would have impaired DDR. This hypothesis is supported by several lines of evidence including the data on the relationship between PFG and DSB in ALL patients [19–21]. Therefore, detailed analysis of DDR in subpopulations of CD34^+^ cells is warranted in an effort to further correlate DDR and PFG formation.

## MATERIALS AND METHODS

### Ethics statement

This study has been approved by the Ethics Committee of Children's Hospital in Bratislava. Human UCB samples were obtained with written parental informed consent. Experiments were carried out *in accordance with* the approved procedure.

### UCB samples

Umbilical cord bloods, 80–100 ml, were collected from 300 newborns by the Eurocord, Bratislava, Slovakia. MNC were isolated from UCB within 24 hours from birth using LymphoSep^TM^ (MP Biomedicals, USA). Number of cells was assessed using autohematology analyzer (Mindray, BC-3000plus, China). Isolated UCB MNC pellets, ~10^7^ MNC, were then frozen and stored in liquid nitrogen before analysis.

### Cell irradiation

UCB cells were irradiated with ^60^Co γ-rays on ice Theratron Elite 100 (Mds Nordion, Ottawa, Ontario, Canada) source.

### RNA isolation and cDNA synthesis

For RNA isolation, a single cell pellet was thawed and total RNA was isolated with RNAzol (Research Molecular Center, Ohio, USA) using standard protocol recommended by the manufacturer. cDNA was synthesized by reverse transcription of total RNA in the standard reaction containing 1 μg of total RNA following the manufacturer's protocol (Thermo Scientific, St. Leon-Rot, Germany) and then used as a template for RT-qPCR.

### Real time quantitative PCR

The RT-qPCR contained 2 μl cDNA (100 ng RNA equivalent), 300 nM each primer, 200 nM probe (5′-fluorophore was FAM, 3′-quencher was BHQ1; synthesized by Merck (Darmstadt, Germany)), and HOT FIREPol Probe qPCR mix from Solis BioDyne (Tartu, Estonia). The primers and probes were synthesized by VBC-Biotech (Wien, Austria) and designed according to Gabert et al. [40]. The plasmid standards with individual fusion genes subcloned into PCR II TOPO vector were from Ipsogen (Qiagen, Marseille, France). RT-qPCR was performed on a BioRad CFX96 instrument following the protocol by Gabert et al. [40]. All precautionary measures taken against contamination have been described previously [14]. All samples were run in triplicate and regarded as positive if at least one of three tested tubes was positive. Positive samples were randomly selected for repeated screening four times while negative samples were repeated twice.

### Sequencing of the RT-qPCR product

The RT-qPCR product was digested with Exo/Sap (Affymetrix, California, USA) to remove all the contaminating primers, TaqMan probe and dNTPs which may interfere with subsequent sequencing. Then the sequencing reaction was performed with the same forward and reverse primers as those used in RT-qPCR assay. If the direct approach did not work, e.g. if the amount of the template was too low, the RT-qPCR product was firstly re-amplified in a standard PCR and then sequenced as mentioned above. Alternatively, the RT-qPCR product was re-amplified in standard PCR using primers that contained restriction sites allowing directed subcloning of the PCR product into a sequencing vector. After the subcloning step, recombinant plasmid DNA was isolated and employed as a template in the sequencing reaction using sequencing primers of the vector.

### DNA repair foci

Cells from ten PFG^+^ and ten matched PFG^−^ probands were analyzed for endogenous DSB using imaging flow cytometry by ImageStream X-100 (Amnis, Seattle, USA) and fluorescence microscopy by the Metafer slide scanning system (MetaSystems, Altlusheim, Germany) as previously described [29]. For the ImageStream analysis, each sample was probed using either polyclonal γH2AX antibody (γH2AXp, Cell Signaling Technology Danvers, MA, USA) in combination with CD34 marker for HSPC (MACS Miltenyi Biotec, Bergisch Gladbach, Germany) or monoclonal γH2AX antibody (γH2AXm, Novus Biologicals, United Kingdom) with polyclonal 53BP1 antibody (Novus Biologicals, United Kingdom). The second combination was used for Metafer analyses. About 500 and 10,000 cells were analyzed for each experimental condition by Metafer and ImageStream, respectively. As a positive control we used induction of γH2AX foci by low doses of γ-rays. We saw statistically significant effect in increasing of γH2AX and 53BP1 foci already at 2 cGy (data not shown).

### Apoptosis

The survival ability of cells was analyzed by FACS by BD FACS Canto™ II cell analyzer using propidium iodide Annexin V-FLUOS staining kit (Roche, Switzerland). Lymphocytes (Ly) and HSPC were gated using the fluorescent specific surface markers and analyzed for % of live, early apoptotic, late apoptotic and necrotic cells. CD45 surface marker (BD Biosciences, San Jose California,USA) was used to distinguish Ly from erythrocytes and debris and CD34 marker (MACS Miltenyi Biotec, Bergisch Gladbach, Germany) was used to measure HSPC. To achieve sufficient statistical power, in average 80,000 MNC (CD45^+^) cells were analyzed at each experimental condition.

### DNA damage response

DNA damage response was detected by alkaline single-cell gel electrophoresis (SCGE) also called comet assay after exposure of MNC to γ-rays at a dose of 3 Gy using THERATRON^®^ Elite 100 (MDS Nordion, Ottawa, Canada). Alkaline SCGE was performed according to the procedure of Gabelova et al. [41]. One hundred EtBr-stained nucleoids were examined per sample with an Axio Imager fluorescence microscope and Metafer 3.6 software (MetaSystems, Altlusheim, Germany). The percentage of DNA in the tail was used as a measure of DNA damage.

### Statistics

Statistical analysis was carried out using Statistica 8.0 (Statsoft, Tulsa, OK). The multivariate Wilks lambda test (ANOVA) was used to estimate variances in PFG^+^ and PFG^−^ groups. Comparison between HSPC and lymphocytes was performed with the two tailed *t*-test. The results were considered significantly different at *p* < 0.05.

## SUPPLEMENTARY MATERIALS FIGURES AND TABLES





## Abbreviations

ALL: acute lymphoblastic leukemia
BM: bone marrow
C_t_: Cycle threshold
DCL: donor-cell derived leukemia
DDR: DNA damage response
dNTPs: deoxynucleotide triphosphates
DSB: double strand break
EtBr: ethidium bromide
HSPC: hematopoietic stem/progenitor cells
MNC: mononuclear cells
PFG: preleukemic fusion gene
PFG^−^: preleukemic fusion gene negative
PFG^+^: preleukemic fusion gene positive
ROS: reactive oxygen species
RT-qPCR: real-time quantitative polymerase chain reaction
UCB: umbilical cord blood
γH2AXm: monoclonal antibody against γH2AX
γH2AXp: polyclonal antibody against γH2AX
53BP1: tumor protein p53 (TP53) binding protein 1
