# Supplementary material for: Low numbers of pre-leukemic fusion genes are frequently present in umbilical cord blood without affecting DNA damage response
Source: Oncotarget. 2017 Mar 15;8(22):35824–34. doi: 10.18632/oncotarget.16211 (PMC5482620; doi:10.18632/oncotarget.16211)
Supplement: Supplementary file 1 [file oncotarget-08-35824-s001.pdf]

Low numbers of pre-leukemic fusion genes are frequently present in umbilical cord blood without affecting DNA damage response

Supplementary Materials

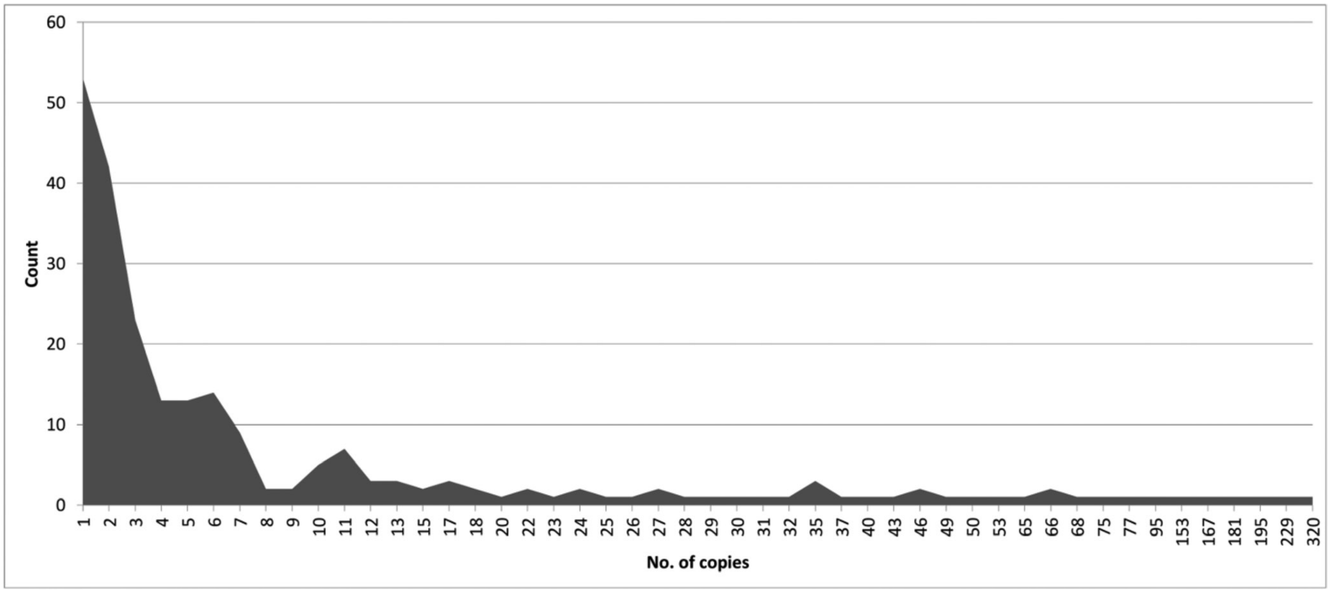

Supplementary Figure 1: Distribution of number of PFG copies analyzed in 272 RT-qPCR runs among 133 PFG-positive probands. The number of PFG copies was usually less than 6 per 100,000 cells. Mean value represents 14 copies.

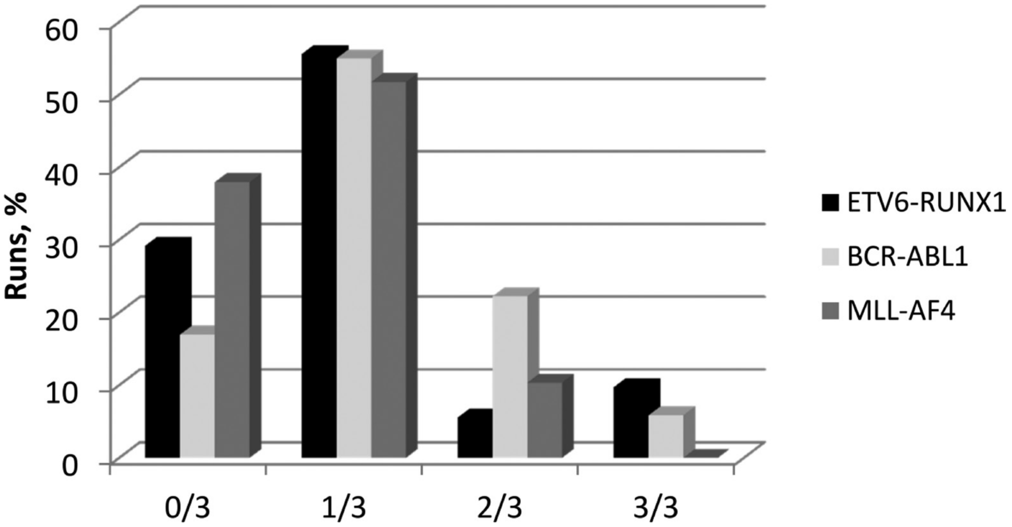

Supplementary Figure 2: Quantitative analysis of 133 positive UCB proband samples (including 90 RT-qPCR repeatedly analyzed samples) obtained by 272 RT-qPCR runs. The percentage of 0/3, 1/3, 2/3 and 3/3 runs, each done in triplicate, for each type of PFG is shown.

Supplementary Table 1: PFG positive UCB MNC samples and PFG negative samples subjected to DDR analyses

Positive (P)	Negative (N)	Sex	Time till processing for (P), h	Time till processing for (N), h
P 143	P 121	M	16	17
P 145	P 127	M	6.5	2
P 218	P 390	F	8	7
P 219	P 539	F	15	14.5
P 230	P 457	M	23	23.5
P 233	P 409	M	19.5	19
P 239	P 336	F	13	13.5
P 310	P 512	F	16	17
P 522	P 529	F	23	22
P 546	P 544	F	19	17.5
Mean ± SD			15.9 ± 5.3	15.3 ± 6.2

Table shows ten PFG positive UCB MNC samples (P) and ten negative samples (N), matched by sex and time of cell processing after birth in hours (h), which were subjected to analysis for endogenous DSB, cell viability, and DDR. M - male; F - female.

Supplementary Data 1: Sequencing data. Summary of positive probands validated by sequencing. See Supplementary_Data_1
