# Supplementary material for: Low numbers of pre-leukemic fusion genes are frequently present in umbilical cord blood without affecting DNA damage response
Source: Oncotarget. 2017 Mar 15;8(22):35824–34. doi: 10.18632/oncotarget.16211 (PMC5482620; doi:10.18632/oncotarget.16211)
Supplement: Supplementary file 2 [file oncotarget-08-35824-s002.docx]

**Supplementary Data S1:** **Sequencing data.** Summary of positive probands validated by sequencing

1. **TEL-AML1:** P143, P216, P218, P219, P230

**I.1a.** # **P143** clone T2(2)

Sequence of the clone (from the sequencing profile):

CCATCCGGCCGTGCCAGCTTGCATGCCTGCAGGTCGACTCTAGAGGATCCAAGGCGGCGTGAAGCGGCGGCTCGTGCTGGCATCGAGGACGTCTCTAGAAGGATTCATTCCAAGTATGCATTCTGCTATTCTCCCAATGGGCATGGCGTGCTCTTCAGGCGGGAATTCCATGGCGTGCTCTTCAGGCGGGAATTCGTAATCATGTCATAGCTGTTTCCTGTGTGAAATTGTTATCCGCTCACAATTCCACACAACATACGAGCCGGAAGCATAAAGTGTAAAGCCTGGGGTGCCTAATGAGTGAGCTAACTCACATTAATTGCGTTGCGCTCACTGCCCGCTTTCCAGTCGGGAAACCTGTCGTGCCAGCTGCATTAATGAATCGGCCAACGCGCGGGGAGAGGCGGTTTGCGTATTGGGCGCTCTTCCGCTTCCTCGCTCACTGACTCGCTGCGCTCGGTCGTTCGGCTGCGGCGAGCGGTATCAGCTCACTCAAAGGCGGTAATACGGTTATCCACAGAATCAGGGGATAACGCAGGAAAGAACATGTGAGCAAAAGGCCAGCAAAAGGCCAGGAACCGTAAAAAGGCCGCGTTGCTGGCGTTTTTCCATAGGCTCCGCCCCCCTGACGAGCATCACAAAAATCGACGCTCAAGTCAGAGGTGGCGAAACCCGACAGGACTATAAAGATACCAGGCGTTTCCCCCTGGAAGCTCCCTCGTGCGCTCTCCTGTTCCGACCCTGCCGCTTACCGGATACCTGTCCGCCTTTCTCCCTTCGGGAAGCGTGGCGCTTTCTCATAGCTCACGCTGTAGGTATCTCAGTTCGGTGTAGGTCGTTCGCTCCAAGCTGGGCTGTGTGCACGAACCCCCCGTTTCAGCCCGACCGCTGCGCCTTATCCGGGTAACTATCGTCTTGAGTCCAACCCGGGTAGACACGACTTATCGCCACTGGCAGCAGCCACTGGTAACAGGATTAAGCAGAGCGAGTATGTAGGCGGTGCCTACAGAGTCTGAGTGTGACCTAACTACGCCTACACTAGAGAACAGTATTTGGTATCTGCGCTCTGCTGGAGCCAGTACTTCGGAAAGAGTGCTAGCTCTGAATCCGGACCA

Alignment of the sequence against published TEL-AML1 fusion transcript (FT) sequence:

**98.0% identity** (98.0% similar) in 98 nt overlap (165-68:8-105)

160 150 140 130 120 110

T2(2) TTCCCGCCTGAAGAGCACGCCATGCCCATTGGGAGAATAGCAGAATGCATACTTGGAATG

: ::::::::::::::::::::::::::::::::::::::::::::::::::::::::::

TEL-AML1 TCCCCGCCTGAAGAGCACGCCATGCCCATTGGGAGAATAGCAGAATGCATACTTGGAATG

10 20 30 40 50 60

100 90 80 70

T2(2) AATCCTTCTAGAGACGTCCTCGATGCCAGCACGAGCCG

::::::::::::::::::: ::::::::::::::::::

TEL-AML1 AATCCTTCTAGAGACGTCCACGATGCCAGCACGAGCCG

70 80 90 100

Translation of the sequence ([3'5' Frame 3](http://web.expasy.org/cgi-bin/translate/dna_sequences?/work/expasy/tmp/http/seqdna.12201,6)):

VRIQS-HSFRSTGSSRAQIPNTVL-CRRS-VTLRLCRHRLHTRSA-SCYQWLLPVAISRV

YPGWTQDDSYPDKAQRSG-NGGFVHTAQLGANDLHRTEIPTA-AMRKRHASRREKGGQVS

GKRQGRNRRAHEGASRGKRLVSL-SCRVSPPLT-ASIFVMLVRGAEPMEKRQQRGLFTVP

GLLLAFCSHVLSCVIP-FCG-PYYRL-VS-YRSPQPNDRAQRVSERGSGRAPNTQTASPR

ALADSLMQLARQVSRLESGQ-AQRN-CELAHSLGTPGFTLYASGSYVVWNCERITISHRK

QL-HDYEFPPEEHAMEFPPEEHAMPIGRIAECILGMNPSRDVLDASTSRRFTPPWIL-SR

PAGMQAGTAGW

Alignment of the sequence against TEL-AML1 fusion protein sequence:

**96.8% identity** (96.8% similar) in 31 aa overlap (303-333:4-34)

310 320 330

T2(2) PPEEHAMPIGRIAECILGMNPSRDVLDASTS

::::::::::::::::::::::::: :::::

TEL-AML1 PPEEHAMPIGRIAECILGMNPSRDVHDASTS

10 20 30

**I.1b.** # **P143** clone T2(11)

Sequence of the clone (between cloning sites, BamHI and HindIII):

CACCGGCAGTGCCAGCTTGCATGCCTGCAGGTCGACTCTAGAGGATCCAAGGCGGCGTGAAGCGGCGGCTCGTGCTGGCATCGTGGACGTCTCTAGAAGGATTCATTCCAAGTATGCATTCTGCTATTCTCCCAATGGGCATGGCGTGCTCTTCAGGCGGGAATTCCATGGCG

Alignment of the sequence against published TEL-AML1 FT sequence:

99.0% identity (99.0% similar) in 98 nt overlap (163-66:8-105)

160 150 140 130 120 110

T2(11) TTCCCGCCTGAAGAGCACGCCATGCCCATTGGGAGAATAGCAGAATGCATACTTGGAATG

: ::::::::::::::::::::::::::::::::::::::::::::::::::::::::::

TEL-AML1 TCCCCGCCTGAAGAGCACGCCATGCCCATTGGGAGAATAGCAGAATGCATACTTGGAATG

10 20 30 40 50 60

100 90 80 70

T2(11) AATCCTTCTAGAGACGTCCACGATGCCAGCACGAGCCG

::::::::::::::::::::::::::::::::::::::

TEL-AML1 AATCCTTCTAGAGACGTCCACGATGCCAGCACGAGCCG

70 80 90 100

Translation of the sequence ([3'5' Frame 2](http://web.expasy.org/cgi-bin/translate/dna_sequences?/work/expasy/tmp/http/seqdna.12201,6)):

AMEFPPEEHAMPIGRIAECILGMNPSRDVHDASTSRRFTPPWIL-SRPAGMQAGTAG

Alignment of the sequence against TEL-AML1 fusion protein sequence:

**100.0% identity** (100.0% similar) in 31 aa overlap (5-35:4-34)

10 20 30

T2(11) PPEEHAMPIGRIAECILGMNPSRDVHDASTS

:::::::::::::::::::::::::::::::

TEL-AML1 PPEEHAMPIGRIAECILGMNPSRDVHDASTS

10 20 30

**I.2.**  # **P216** clone T2(1)

Sequence of the clone (in bold between cloning sites, BamHI and HindIII):

CCTCGGCCAGTGCAGCTTGCATGCCTGCAGGTCGACTCTAGAGGATCC**AAGGCGGCGTGAAGCGGCGGCTCGTGCTGGCATCGTGGACGTCTCTAGAAGGATTCATTCCAAGTATGCATTCTGCTATTCTCCCAATGGGCATGGCGTGCTCTTCAGGCGG**GAATTCGTAATCATGTCATA

GCTGTTTCCTGTGTGAAATTGTTATCCGCTCACAATTCCACACAACATACGAGCCGGAAGCATAAAGTGTAAAGCCTGGGGTGCCTAATGAGTGAGCTAACTCACATTAATTGCGTTGCGCTCACTGCCCGCTTTCCAGTCGGGAAACCTGTCGTGCCAGCTGCATTAATGAATCGGCCA

ACGCGCGGGGAGAGGCGGTTTGCGTATTGGGCGCTCTTCCGCTTCCTCGCTCACTGACTCGCTGCGCTCGGTCGTTCGGCTGCGGCGAGCGGTATCAGCTCACTCAAAGGCGGTAATACGGTTATCCACAGAATCAGGGGATAACGCAGGAAAGAACATGTGAGCAAAAGGCCAGCAAAA

GGCCAGGAACCGTAAAAAGGCCGCGTTGCTGGCGTTTTTCCATAGGCTCCGCCCCCCTGACGAGCATCACAAAAATCGACGCTCAAGTCAGAGGTGGCGAAACCCGACAGGACTATAAAGATACCAGGCGTTTCCCCCTGGAAGCTCCCTCGTGCGCTCTCCTGTTCCGACCCTGCCGCT

TACCGGATACCTGTCCGCCTTTCTCCCTTTCGGAAAGCGTGCGCTTTCTCATAGCTCACGCTGTAGGTATCTCAGTTCGGTGTAGGTCGTTCGCCTCCAAGCTGGCTGTGTGCACGACCCCCCGTTCAGCCCGACCGCTGCGCTTATCGGTACTATCGTCTGAGTCACCGGTAAGACACG

ACTTATCGCACTGCAGCAGCCACTGTTACAGATAAGCAGAGCGAGTTATGTAGCCGTGCTACAGAGTCTGAATGATGACCTACTACGGCTACCTAGAAGACGTATTGAATCTGCCTGCTGAAGCAGTACTCGAGGATGTAGCTCTTGA

Alignment of the sequence against published TEL-AML1 FT sequence:

**99.0% identity** (99.0% similar) in 98 nt overlap (163-66:8-105)

160 150 140 130 120 110

T2(1) TTCCCGCCTGAAGAGCACGCCATGCCCATTGGGAGAATAGCAGAATGCATACTTGGAATG

: ::::::::::::::::::::::::::::::::::::::::::::::::::::::::::

TEL-AML1 TCCCCGCCTGAAGAGCACGCCATGCCCATTGGGAGAATAGCAGAATGCATACTTGGAATG

10 20 30 40 50 60

100 90 80 70

T2(1) AATCCTTCTAGAGACGTCCACGATGCCAGCACGAGCCG

::::::::::::::::::::::::::::::::::::::

TEL-AML1 AATCCTTCTAGAGACGTCCACGATGCCAGCACGAGCCG

70 80 90 100

Translation of the insert ([3'5' Frame 1](http://web.expasy.org/cgi-bin/translate/dna_sequences?/work/expasy/tmp/http/seqdna.12201,6)):

PPEEHAMPIGRIAECILGMNPSRDVHDASTSRRFTPP

Alignment of the sequence against TEL-AML1 fusion protein sequence:

**100.0% identity in 31 aa overlap** (1-31:4-34); score: 218 E(10000): 2.5e-17

10 20 30

T2(1) PPEEHAMPIGRIAECILGMNPSRDVHDASTS

:::::::::::::::::::::::::::::::

TEL-AML1 PPEEHAMPIGRIAECILGMNPSRDVHDASTS

10 20 30

**I.3a.** # **P218** clone T3(1)

Sequence of the clone (in bold between cloning sites, BamHI and HindIII):

CCTCGCAGTGCAGCTTGCATGCCTGCAGGTCGACTCTAGAGGATCC**AAGGCGGCGTGAAGCGGCGGGATCTAAGGCGGCGTGAAGCGGCGGCTCGTGCTGGCATGGTTCAGGCGGGGAGACAGAGAGATTCATCCAAGTATGCATTCTGCTATTCTCCCAATGGGCATGGCGTGCTCTTCAGGCGG**GAATTCGTAATCATGTCATAGCTGTTTCCTGTGTGAAATTGTTATCCGCTCACAATTCCACACAACATACGAGCCGGAAGCATAAAGTGTAAAGCCTGGGGTGCCTAATGAGTGAGCTAACTCACATTAATTGCGTTGCGCTCACTGCCCGCTTTCCAGTCGGGAAACCTGTCGTGCCAGCTGCATTAATGAATCGGCCAACGCGCGGGGAGAGGCGGTTTGCGTATTGGGCGCTCTTCCGCTTCCTCGCTCACTGACTCGCTGCGCTCGGTCGTTCGGCTGCGGCGAGCGGTATCAGCTCACTCAAAGGCGGTAATACGGTTATCCACAGAATCAGGGGATAACGCAGGAAAGAACATGTGAGCAAAAGGCCAGCAAAAGGCCAGGAACCGTAAAAAGGCCGCGTTGCTGGCGTTTTTCCATAGGCTCCGCCCCCCTGACGAGCATCACAAAAATCGACGCTCAAGTCAGAGGTGGCGAAACCCGACAGGACTATAAAGATACCAGGCGTTTCCCCCTGGAAAGCTCCCTCGTGCGCTCTCCTGTTCCGACCCTGCCGCTTACCGGATACCTGTCCGCCTTTCTCCCTTCGGGAAGCGTGCGCTTTCTCATAGCTCACGCTGTAGGTATCTCAGTTCGGTGTAGGTCGTTCGCCTCCAAGCTGGGCTGTGTGCACGAACCCCCCCGTTCAGCCCGACCGCTGCGCTTATCCGGTAACTATCGTCTTGAGTCAACCGGTAAGAACACGACTTATCGCCACTGCAGCAGCAACTGTACAGATTAGCAGAGCGAGTATGTAGCGTGCTACAGAGTTCTTGGATTGCTGCTACCTACGGCTACCTAGAAGAAACTGTATTCGATCTGCCGTCTAGCTGAAGCTGGTTACCTTACCGA

Alignment of the sequence against published TEL-AML1 FT sequence:

**82.9% identity** (82.9% similar) in 105 nt overlap (189-89:8-105)

190 180 170 160 150 140

T3(1) TTCCCGCCTGAAGAGCACGCCATGCCCATTGGGAGAATAGCAGAATGCATACTTGGA-TG

: ::::::::::::::::::::::::::::::::::::::::::::::::::::::: ::

TEL-AML1 TCCCCGCCTGAAGAGCACGCCATGCCCATTGGGAGAATAGCAGAATGCATACTTGGAATG

10 20 30 40 50 60

130 120 110 100 90

T3(1) AATCTCTCT---GTCTCCCCGCCTGAACCATGCCAGCACGAGCCG

:::: ::: : : :: :: ::::::::::::::::

TEL-AML1 AATCCTTCTAGAGACGTCC-------ACGATGCCAGCACGAGCCG

70 80 90 100

Translation of the insert ([3'5' Frame 1](http://web.expasy.org/cgi-bin/translate/dna_sequences?/work/expasy/tmp/http/seqdna.12201,6)):

PPEEHAMPIGRIAECILG-ISLSPRLNHASTSRRFTPP-IPPLHAA

Alignment of the sequence against TEL-AML1 fusion protein sequence:

**74.2% identity in 31 aa overlap** (1-31:4-34); score: 151 E(10000): 1.9e-10

10 20 30

T3(1) PPEEHAMPIGRIAECILGISLSPRLNHASTS

::::::::::::::::::.. : .. ::::

TEL-AM PPEEHAMPIGRIAECILGMNPSRDVHDASTS

10 20 30

**I.3b.** # **P218** clone T3(12)

Sequence of the clone (from the sequencing profile):

CAGGAATGCTTACGCCAGCTCTAATACGACTCACTATAGGGAAAGCTTGCATGCAGGCCTCTGCAGTCGACGGGCCCGGGATCCGATTCGGGATCCAAGGCGCGTGAAGCGGCGGCTCGTGCTGGCATCGTGGACGTCTCTAGAAGGGTTCATTCCAAGTATGCATTCTGCTATTCTCCCAATGGGCATGGCGTGCTCTTCAGGCGGGAATTCCAATCTAGATGCATTCGCGAGGTACCGAGCTCGAATTCACTGGCCGTCGTTTTACAACGTCGTGACTGGGAAAACCCTGGCGTTACCCAACTTAATCGCCTTGCAGCACATCCCCCTTTCGCCAGCTGGCGTAATAGCGAARAGGCCCGCACCGATCGCCCTTCCCAACAGTTGCGCAGCCTGAATGGCGAATGGAAATTGTAAGCGTTAATATTTTGTTAAAATTCGCGTTAAATTTTTGTTAAATCAGCTCATTTTTTAACCAATAGGCCGAAATCGGCAAAATCCCTTATAAATCAAAAGAATAGACCGAGATAGGGTTGAGTGTTGTTCCAGTTTGGAACAAGAGTCCACTATTAAAGAACGTGGACTCCAACGTCAAAGGGCGAAAAACCGTCTATCAGGGCGATGGCCCACTACGTGAACCATCACCCTAATCAAGTTTTTTGGGGTCGAGGTGCCGTAAAGCACTAAATCGGAACCCTAAAGGGAGCCCCCGATTTAGAGCTTGACGGGGAAAGCCGGCGAACGTGGCGAGAAAGGAAGGGAAGAAAGCGAAAGGAGCGGGCGCTAGGGCGCTGGCAAGTGTAGCGGTCACGCTGCGCGTAACCACCACACCCGCCGCGCTTAATGCGCCGCTACAGGGCGCGTCAGGTGGCACTTTTTCGGGGAAATGTGCGCGGAACCCCTATTTGTTTATTTTTCTAAATACATTCAAATATGTATCCGCTCATGAGACAATAACCCTGATAAATGCTTCATAATATTGAAAAGGGAGAGTATGAGTATTCCACATTTTCGTGTCGCCATATCGTTTTTTGCCGCATTGCCGTCCTGTTTTGCTCACCAGAACCGCCTTGGT

**98.0% identity** (98.0% similar) in 98 nt overlap (210-113:8-105)

210 200 190 180 170 160

T3(12) TTCCCGCCTGAAGAGCACGCCATGCCCATTGGGAGAATAGCAGAATGCATACTTGGAATG

: ::::::::::::::::::::::::::::::::::::::::::::::::::::::::::

TEL-AML1 TCCCCGCCTGAAGAGCACGCCATGCCCATTGGGAGAATAGCAGAATGCATACTTGGAATG

10 20 30 40 50 60

150 140 130 120

T3(12) AACCCTTCTAGAGACGTCCACGATGCCAGCACGAGCCG

:: :::::::::::::::::::::::::::::::::::

TEL-AML1 AATCCTTCTAGAGACGTCCACGATGCCAGCACGAGCCG

70 80 90 100

Translation of the sequence ([3'5' Frame 2](http://web.expasy.org/cgi-bin/translate/dna_sequences?/work/expasy/tmp/http/seqdna.12201,6)):

PRRFW-AKQDGNAAKNDMATRKCGILILSLFNIMKHLSGLLSHERIHI-MYLEK-TNRGS

AHISPKKCHLTRPVAAH-ARRVWWLRAA-PLHLPAP-RPLLSLSSLPFSPRSPAFPVKL-

IGGSL-GSDLVLYGTSTPKNLIRVMVHVVGHRPDRRFFAL-RWSPRSLIVDSCSKLEQHS

TLSRSILLIYKGFCRFRPIG-KMS-FNKNLTRILTKY-RLQFPFAIQAAQLLGRAIGAGX

FAITPAGERGMCCKAIKLGNARVFPVTTL-NDGQ-IRARYLANASRLEFPPEEHAMPIGR

IAECILGMNPSRDVHDASTSRRFTRLGSRIGSRARRLQRPACKLSL--VVLELA-AFL

Alignment of the sequence against TEL-AML1 fusion protein sequence:

**100.0% identity in 31 aa overlap (276-306:4-34); score: 218 E(10000):** 2.4e-16

280 290 300

T3(12) PPEEHAMPIGRIAECILGMNPSRDVHDASTS

:::::::::::::::::::::::::::::::

TEL-AM PPEEHAMPIGRIAECILGMNPSRDVHDASTS

10 20 30

**I.4a.** # **P219** clone T3(1)

Sequence of the clone (from the sequencing profile):

CATTAMTGCATTACGCCAGCTCTAATACGACTCACTATAGGGAAAGCTTGCATGCAGGCCTCTGCAGTCGACGGGCCCGGGATCCGATTCGGCTCGTGCTGGCATCGTGGACGTCTCTAGAAGGATTCATTCCAAGTATGCATTCTGCTATTCTCCCAATGGGCATGGCGTGCTCTTCAGGCGGGGAGACAGAGAATCTAGATGCATTCGCGAGGTACCGAGCTCGAATTCACTGGCCGTCGTTTTACAACGTCGTGACTGGGAAAACCCTGGCGTTACCCAACTTAATCGCCTTGCAGCACATCCCCCTTTCGCCAGCTGGCGTAATAGCGAAGAGGCCCGCACCGATCGCCCTTCCCAACAGTTGCGCAGCCTGAATGGCGAATGGAAATTGTAAGCGTTAATATTTTGTTAAAATTCGCGTTAAATTTTTGTTAAATCAGCTCATTTTTTAACCAATAGGCCGAAATCGGCAAAATCCCTTATAAATCAAAAGAATAGACCGAGATAGGGTTGAGTGTTGTTCCAGTTTGGAACAAGAGTCCACTATTAAAGAACGTGGACTCCAACGTCAAAGGGCGAAAAACCGTCTATCAGGGCGATGGCCCACTACGTGAACCATCACCCTAATCAAGTTTTTTGGGGTCGAGGTGCCGTAAAGCACTAAATCGGAACCCTAAAGGGAGCCCCCGATTTAGAGCTTGACGGGGAAAGCCGGCGAACGTGGCGAGAAAGGAAGGGAAGAAAGCGAAAGGAGCGGGCGCTAGGGCGCTGGCAGTGTAGCGGTCACGCTGCGCGTACCACACCACCGCGCGCTTAATGCGCCGCTACAGGCGCGTCAGGTGGCACTTTTCGGGAATGTGSGCGACCCCTATTGTTATTTTTCTAATACATCAATATGTWTCCGCTCATGAGACAATACCTGATAATGCTTCATAATGAAAATGAGAGTTGRTATCGACTTTCGGTTCGCTATTATTGCGCACTTGCATCGGTATG

Alignment of the sequence against published TEL-AML1 FT sequence:

**100.0% identity** (100.0% similar) in 105 nt overlap (194-90:1-105)

190 180 170 160 150 140

T3(1) CTCTGTCTCCCCGCCTGAAGAGCACGCCATGCCCATTGGGAGAATAGCAGAATGCATACT

::::::::::::::::::::::::::::::::::::::::::::::::::::::::::::

TEL-AML1 CTCTGTCTCCCCGCCTGAAGAGCACGCCATGCCCATTGGGAGAATAGCAGAATGCATACT

10 20 30 40 50 60

130 120 110 100

T3(1) TGGAATGAATCCTTCTAGAGACGTCCACGATGCCAGCACGAGCCG

:::::::::::::::::::::::::::::::::::::::::::::

TEL-AML1 TGGAATGAATCCTTCTAGAGACGTCCACGATGCCAGCACGAGCCG

70 80 90 100

Translation of the sequence ([3'5' Frame 3](http://web.expasy.org/cgi-bin/translate/dna_sequences?/work/expasy/tmp/http/seqdna.12201,6)):

YRCKCAIIANRKSIXTLIFIMKHYQVLSHERXHIDVLEK-Q-GSXTFPKSAT-RACSGAL

SARWCGTRSVTATLPAP-RPLLSLSSLPFSPRSPAFPVKL-IGGSL-GSDLVLYGTSTPK

NLIRVMVHVVGHRPDRRFFAL-RWSPRSLIVDSCSKLEQHSTLSRSILLIYKGFCRFRPI

G-KMS-FNKNLTRILTKY-RLQFPFAIQAAQLLGRAIGAGLFAITPAGERGMCCKAIKLG

NARVFPVTTL-NDGQ-IRARYLANASRFSVSPPEEHAMPIGRIAECILGMNPSRDVHDAS

TSRIGSRARRLQRPACKLSL--VVLELA-CXN

Alignment of the sequence against TEL-AML1 fusion protein sequence:

**100.0% identity in 34 aa overlap** (257-290:1-34); score: 233 E(10000): 7.1e-18

260 270 280 290

T3(1) SVSPPEEHAMPIGRIAECILGMNPSRDVHDASTS

::::::::::::::::::::::::::::::::::

TEL-AML1 SVSPPEEHAMPIGRIAECILGMNPSRDVHDASTS

10 20 30

**I.4b.** # **P219** clone T3(4)

Sequence of the clone (from the sequencing profile):

GAGKGAATCATTWCGCCAGCTCTATACGACTCACTATAGGGAAAGCTTGCATGCAGGCCTCTGCAGTCSACGGGCCCGGGATCCGATTCGGGATCCAAGGCGGCGTGAAGGGSGGCTCGKGSTGGCATCGTGGACGTCTCTAGAAGGATTCWTTCCAAGTATGCATTCTGCTATTCTCCCAATGGGCATGGSGTGCTCTTCAGGCGGGAATTCCAATCTAGATGCATTCGCGAGGTACCGAGCTCGAATTCACTGGCCGTCGTTTTACAACGTCGTGACTGGGAAAACCCTGGCGTTACCCAACTTAATCGCCTTGCRGCACATCCCCCTTTCGCCAGCTGGSGTAATAGCGAARAGGCCCGCACCGATCGCCCTTCCCAACAGTTGCGCASCCTGAATGGSGAATGGAAATTGTAAGCGTTAATATTTTGTTAAAATTCGCGTTAAATTTTTGTTAAATCASCTCATTTTTTAACCAATAGGCCGAAATCGGCAAAATCCCTTATAAATCAAAARAATAGACCGAGATAGGGTTGAGTGTKGTTCCAGTTTGGAACAAGAGTCCACTATTAAAGAACGTGGACTCCAMCGTCAAAGGGCGAAAAACCGTCTATCAGGGCGATGGCCCACTACGTGAACCATCACCCTAATCAAGTTTTTTGGGGTCGAGGTGCCGTAAAGCACTAAATCGGAACCCTAAAGGGAGCCCCCGATTTAGAGCTTGASGGGGAAAGCCGGCGAACGTGGCGAGAAAGGAAGGGAAGAAAGCGAAAGGAGCGGGCGCTAGGGCGCTGGCAAGTGTAGCGGTCACGCTGCGCGTAACCACCACACCCGCCGCGCTTAATGCGCCGCTACAGGGCGCGTCAGGTGGGCACTTTTCGGGGGAAATGTGCGCGGAACCCCTATTTGTKTATTTTTTCTAAATACATTCAAATATGTATCCGCTCATGAGACAATAACCCTGATAAATGCTTCATTATATTGAAAAGGGAGAGTATGAGTATCACATTTCCGTGTCGTCATATTCCTTTTTGCGCCATTTGCCTTCCTGTTTTTGCTCACCCAGAAACGGC

Alignment of the sequence against published TEL-AML1 FT sequence:

**93.9% identity** (99.0% similar) in 98 nt overlap (210-113:8-105)

210 200 190 180 170 160

T3(4) TTCCCGCCTGAAGAGCACSCCATGCCCATTGGGAGAATAGCAGAATGCATACTTGGAAWG

: ::::::::::::::::.:::::::::::::::::::::::::::::::::::::::.:

TEL-AML1 TCCCCGCCTGAAGAGCACGCCATGCCCATTGGGAGAATAGCAGAATGCATACTTGGAATG

10 20 30 40 50 60

150 140 130 120

T3(4) AATCCTTCTAGAGACGTCCACGATGCCASCMCGAGCCS

::::::::::::::::::::::::::::.:.::::::.

TEL-AML1 AATCCTTCTAGAGACGTCCACGATGCCAGCACGAGCCG

70 80 90 100

Translation of the sequence ([3'5' Frame 3](http://web.expasy.org/cgi-bin/translate/dna_sequences?/work/expasy/tmp/http/seqdna.12201,6)):

RFWVSKNRKANGAKRNMTTRKCDTHTLPFQYNEAFIRVIVS-ADTYLNVFRKNIQIGVPR

TFPPKSAHLTRPVAAH-ARRVWWLRAA-PLHLPAP-RPLLSLSSLPFSPRSPAFPXKL-I

GGSL-GSDLVLYGTSTPKNLIRVMVHVVGHRPDRRFFAL-XWSPRSLIVDSCSKLELHST

LSRSIXLIYKGFCRFRPIG-KMX-FNKNLTRILTKY-RLQFPFXIQXAQLLGRAIGAGXF

AIXPAGERGMCXKAIKLGNARVFPVTTL-NDGQ-IRARYLANASRLEFPPEEHXMPIGRI

AECILGXNPSRDVHDAXSSXPSRRLGSRIGSRARXLQRPACKLSL--VV-SWXNDSL

Alignment of the sequence against TEL-AML1 fusion protein sequence:

**87.1% identity in 31 aa overlap (277-307:4-34); score: 195 E(10000): 5e-**14

280 290 300

T3(4) PPEEHXMPIGRIAECILGXNPSRDVHDAXSS

::::: :::::::::::: ::::::::: .:

TEL-AM PPEEHAMPIGRIAECILGMNPSRDVHDASTS

10 20 30

**I.5.** # **P230** clone T5(3)

Sequence of the clone between cloning sites, BamHI and HindIII:

CAGAGGGCAGTGCCAGCTTGCATGCCTGCAGGTCGACTCTAGAGGATCCAAGGCGGCGTGAAGCGGCGGCTCGTGCTGGCATCGTGGACGTCTCTAGAAGGATTCATTCCAAGTATGCATTCTGCTATTCTCCCAATGGGCATGGCGT

Alignment of the sequence against published TEL-AML1 FT sequence:

**100.0% identity** (100.0% similar) in 82 nt overlap (148-67:24-105)

140 130 120 110 100 90

T5(3) ACGCCATGCCCATTGGGAGAATAGCAGAATGCATACTTGGAATGAATCCTTCTAGAGACG

::::::::::::::::::::::::::::::::::::::::::::::::::::::::::::

TEL-AML1 ACGCCATGCCCATTGGGAGAATAGCAGAATGCATACTTGGAATGAATCCTTCTAGAGACG

30 40 50 60 70 80

80 70

T5(3) TCCACGATGCCAGCACGAGCCG

::::::::::::::::::::::

TEL-AML1 TCCACGATGCCAGCACGAGCCG

1. 100

Translation of the insert ([3'5' Frame 3](http://web.expasy.org/cgi-bin/translate/dna_sequences?/work/expasy/tmp/http/seqdna.12201,6)):

AMPIGRIAECILGMNPSRDVHDASTSRRFTPPWIL-SRPAGMQAGTAL

Alignment of the sequence against TEL-AML1 fusion protein sequence:

**100.0% identity** (100.0% similar) in 31 aa overlap (5-35:4-34)

10 20 30

T5(3) PPEEHAMPIGRIAECILGMNPSRDVHDASTS

:::::::::::::::::::::::::::::::

TEL-AML1 PPEEHAMPIGRIAECILGMNPSRDVHDASTS

10 20 30

……………………………………………………………………………………………………………………………………………………………………………………………………

**II. BCR-ABL (p190):** P141, P143, P145, P217, P233, P522, P546,

P377, P138, P203, P206, P215, P239, P310

**II.1a.** # **P141** clone B1

Sequence of the clone (with M13/pUC-R sequencing primer):

CCTCGGCAGTGCAGCTTCACGATGGCGAGGGCGCCTTCCATGGAGACGCAAAAGCCCTTCAGCGGCCAGTAGCATCTGACCTTGAGCCTTCAGGGTCTGAGGGATCCCCGGGTACCGAGCTCGAATTCGTAATCATGTCATAGCTGTTTCCTGTGTGAAATTGTTATCCGCTCACAATTCCACACAACATACGAGCCGGAAGCATAAAGTGTAAAGCCTGGGGTGCCTAATGAGTGAGCTAACTCACATTAATTGCGTTGCGCTCACTGCCCGCTTTCCAGTCGGGAAACCTGTCGGGCCAGCTGCATTAATGAATCGGCCAACGCGCGGGGAGAGGCGGTTTGCGTATTGGGCGCTCTTCCGCTTCCTCGCTCACTGACTCGCTGCGCTCGGTCGTTCGGCTGCGGCGAGCGGTATCAGCTCACTCAAAGGCGGTAATACGGTTATCCACAGAATCAGGGGATAACGCAGGAAAGAACATGTGAGCAAAAGGCCAGCAAAAGGCCAGGAACCGTAAAAAGGCCGCGTTGCTGGCGTTTTTCCATAGGCTCCGCCCCCCTGACGAGCATCACAAAAATCGACGCTCAAGTCAGAGGTGGCGAAACCCGACAGGACTATAAAGATACCAGGCGTTTCCCCCTGGAAGCTCCCTCGTGCGCTCTCCTGTTCCGACCCTGCCGCTTACCGGATACCTGTCCGCCTTTCTCCCTTCGGGAAGCGTGCGCTTTCTCATAGCTCACGCTGTAGGTATCTCAGTTCGGTGTAGGTCGTTCGCCTCCAAGCTGGGCTGTGTGCACGACCCCCCGTTCAGCCCGACCGCTGCGCTTATCCGGGTACTATCGTCTTGAGTCCACCCGGTAGACACGACTTATCCGCCACTGGCAGCAGCCACTGTACAGGAATTAGCAGAGCGAGTATGTAGCGTGCTACAGAGTTCTGAAGTGGTTGCTAACCTACCGCTACCTTAGAGACGTATTGCTATCTGCGCTCTGCTGAAGCAGTACGTCGGTAAAGAGTGGTAGCTCTGATCGCATCAACACGCTTGGTACCGGTGA

Alignment of the sequence against published BCR-ABL (p190) FT sequence:

**95.3% identity** (95.3% similar) in 85 nt overlap (19-103:9-92)

20 30 40 50 60 70

B1 ACGATGGCGAGGGCGCCTTCCATGGAGACGCAAAAGCCCTTCAGCGGCCAGTAGCATCTG

:::::::::::::::::::::::::::::::: :::::::::::::::::::::::::::

BCR-ABL ACGATGGCGAGGGCGCCTTCCATGGAGACGCAGAAGCCCTTCAGCGGCCAGTAGCATCTG

10 20 30 40 50 60

80 90 100

B1 ACCTTGAGCCTTCAGGGTCTGAGGG

:: :::::::: ::::::::::: :

BCR-ABL ACTTTGAGCCT-CAGGGTCTGAGTG

70 80 90

Sequence from START to BamHI-cloning site:

CCTCGGCAGTGCAGCTTCACGATGGCGAGGGCGCCTTCCATGGAGACGCAAAAGCCCTTCAGCGGCCAGTAGCATCTGACCTTGAGCCTTCAGGGTCTGAG

Translation of the sequence ([5'3' Frame 3](http://web.expasy.org/cgi-bin/translate/dna_sequences?/work/expasy/tmp/http/seqdna.13933,3)):

SAVQLHDGEGAFHGDAKALQRPVASDLEPSGSE

Alignment of the sequence against BCR-ABL (p190) fusion protein sequence:

**84.6% identity in 26 aa overlap** (6-31:3-28); score: 158 E(10000): 1.6e-11

10 20 30

B1 HDGEGAFHGDAKALQRPVASDLEPSG

.::::::::::.:::::::::.::.:

BCR-ABL (p190) NDGEGAFHGDAEALQRPVASDFEPQG

10 20

**II.1b.** # **P141** clone B1

Sequence of the clone (with M13/pUC-F sequencing primer):

CAGTACATGATTACGATTCGAGCTCGGTACCCGGGGATCCCTCAGACCCTGAAGGCTCAAGGTCAGATGCTACTGGCCGCTGAAGGGCTTCTGCGTCTCCATGGAAGGCGCCCTCGCCATCGTTGAAGCTTGGCACTGGCCGTCGTTTTACAACGTCGTGACTGGGAAAACCCTGGCGTTACCCAACTTAATCGCCTTGCAGCACATCCCCCTTTCGCCAGCTGGCGTAATAGCGAAGAGGCCCGCACCGATCGCCCTTCCCAACAGTTGCGCAGCCTGAATGGCGAATGGCGCCTGATGCGGTATTTTCTCCTTACGCATCTGTGCGGTATTTCACACCGCATATGGTGCACTCTCAGTACAATCTGCTCTGATGCCGCATAGTTAAGCCAGCCCCGACACCCGCCAACACCCGCTGACGCGCCCTGACGGGCTTGTCTGCTCCCGGCATCCGCTTACAGACAAGCTGTGACCGTCTCCGGGAGCTGCATGTGTCAGAGGTTTTCACCGTCATCACCGAAACGCGCGAGACGAAAGGGCCTCGTGATACGCCTATTTTTATAGGTTAATGTCATGATAATAATGGTTTCTTAGACGTCAGGTGGCACTTTTCGGGGAAATGTGCGCGGAACCCCTATTTGTTTATTTTTCTAAATACATTCAAATATGTATCCGCTCATGAGACAATAACCCTGATAAATGCTTCAATAATATTGAAAAAGGAAGAGTATGAGTATTCAACATTTCCGTGTCGCCCTTATTCCCTTTTTTGCGGCATTTTGCCTTCCTGTTTTTGCTCACCCAGAAACGCTGGTGAAAGTAAAAGATGCTGAAGATCAGTTGGGTGCACGAGTGGGTTACATCGAAACTGGATCTCAACAGCGGTAAGATCCTTGAGAGTTTTCGCCCCGAAGAACGTTTTCCAATGATGAGCACTTTTAAAGTTCTGCTATGTGGCGCGGTATTATCCCGTATTGACGCCGGGCAAGAGCACTTCGGTCGGCCGGCATACACTATTCTCAGATTGAACTGTGAGTACTCCACCAGTCACAGAAAAGCATCTTAACGGGAATGGCATGACAGTAGAGTATATGGCAGTGCCTGCCATAATCCAAG

Alignment of the sequence against published BCR-ABL (p190) FT sequence:

**96.6% identity** (96.6% similar) in 87 nt overlap (125-39:7-92)

120 110 100 90 80 70

B1 CAACGATGGCGAGGGCGCCTTCCATGGAGACGCAGAAGCCCTTCAGCGGCCAGTAGCATC

::::::::::::::::::::::::::::::::::::::::::::::::::::::::::::

BCR-ABL CAACGATGGCGAGGGCGCCTTCCATGGAGACGCAGAAGCCCTTCAGCGGCCAGTAGCATC

10 20 30 40 50 60

60 50 40

B1 TGACCTTGAGCCTTCAGGGTCTGAGGG

:::: :::::::: ::::::::::: :

BCR-ABL TGACTTTGAGCCT-CAGGGTCTGAGTG

70 80 90

Translation of the sequence ([3'5' Frame 3](http://web.expasy.org/cgi-bin/translate/dna_sequences?/work/expasy/tmp/http/seqdna.8623,6)):

WIMAGTAIYSTVMPFPLRCFSVTGGVLTVQSENSVCRPTEVLLPGVNTG-YRAT-QNFKS

AHHWKTFFGAKTLKDLTAVEIQFRCNPLVHPTDLQHLLLSPAFLGEQKQEGKMPQKRE-G

RHGNVEYSYSSFFNIIEAFIRVIVS-ADTYLNVFRKINK-GFRAHFPEKCHLTSKKPLLS

-H-PIKIGVSRGPFVSRVSVMTVKTSDTCSSRRRSQLVCKRMPGADKPVRARQRVLAGVG

AGLTMRHQSRLY-ECTICGVKYRTDA-GENTASGAIRHSGCATVGKGDRCGPLRYYASWR

KGDVLQGD-VG-RQGFPSHDVVKRRPVPSFNDGEGAFHGDAEALQRPVASDLEPSGSEGS

PGTELES-SCT

Alignment of the sequence against BCR-ABL (p190) fusion protein sequence:

**92.3% identity** (100.0% similar) in 26 aa overlap (320-345:3-28)

320 330 340

B1 NDGEGAFHGDAEALQRPVASDLEPSG

:::::::::::::::::::::.::.:

BCR-ABL NDGEGAFHGDAEALQRPVASDFEPQG

10 20

**II.2a.** # **P144** clone B2

Sequence of the clone (with M13/pUC-R sequencing primer):

CATCGGGCAGTGCCAGCTTCACGATGGCGAGGGCGCCTTCCATGGAGACGCAAAAGCCCTTCAGCGGCCAGTAGCATCTGACTTTGAGCCTTCAGGGTCTGAGGGATCCCCGGGTACCGAGCTCGAATTCGTAATCATGTCATAGCTGTTTCCTGTGTGAAATTGTTATCCGCTCACAATTCCACACAACATACGAGCCGGAAGCATAAAGTGTAAAGCCTGGGGTGCCTAATGAGTGAGCTAACTCACATTAATTGCGTTGCGCTCACTGCCCGCTTTCCAGTCGGGAAACCTGTCGTGCCAGCTGCATTAATGAATCGGCCAACGCGCGGGGAGAGGCGGTTTGCGTATTGGGCGCTCTTCCGCTTCCTCGCTCACTGACTCGCTGCGCTCGGTCGTTCGGCTGCGGCGAGCGGTATCAGCTCACTCAAAGGCGGTAATACGGTTATCCACAGAATCAGGGGATAACGCAGGAAAGAACATGTGAGCAAAAGGCCAGCAAAAGGCCAGGAACCGTAAAAAGGCCGCGTTGCTGGCGTTTTTCCATAGGCTCCGCCCCCCTGACGAGCATCACAAAAATCGACGCTCAAGTCAGAGGTGGCGAAACCCGACAGGACTATAAAGATACCAGGCGTTTCCCCCTGGAAGCTCCCTCGTGCGCTCTCCTGTTCCGACCCTGCCGCTTACCGGATACCTGTCCGCCTTTTCTCCCTTCGGGAAGCGTGCGCTTTCTCATAGCTCACGCTGTAGGTATCTCAGTTCGGTGTAGGTCGTTCGCCTCCAAGCTGGGCTGTGTGCACGAACCCCCCGTTTCAGCCCGACCGCTGCGCTTATCCGGTAACTATCGTCTTGAGTCCAACCGGTAAGACACGACTTATCGCCACTGGCAGCAGCCACTGGTAACAGGATTAGCAGAGCGAGTATGTAGGCGGTGCTACAGAGTTCTTGAAGTGGTTGCCTACCTACCGGCCTACCTAGAGAACAGTATTTGCTATCTGCGCCTCTGCTGAAGCAAGTACGTCGGAAATGATTGGTAGCTCTTGGATCGCTACGAGCCACTGCTGGTAACGGTGTACTATG

Alignment of the sequence against published BCR-ABL (p190) FT sequence:

**96.5% identity** (96.5% similar) in 85 nt overlap (21-105:9-92)

30 40 50 60 70 80

B2 ACGATGGCGAGGGCGCCTTCCATGGAGACGCAAAAGCCCTTCAGCGGCCAGTAGCATCTG

:::::::::::::::::::::::::::::::: :::::::::::::::::::::::::::

BCR-ABL ACGATGGCGAGGGCGCCTTCCATGGAGACGCAGAAGCCCTTCAGCGGCCAGTAGCATCTG

10 20 30 40 50 60

90 100

B2 ACTTTGAGCCTTCAGGGTCTGAGGG

::::::::::: ::::::::::: :

BCR-ABL ACTTTGAGCCT-CAGGGTCTGAGTG

70 80 90

Sequence from START to BamHI-cloning site:

CATCGGGCAGTGCCAGCTTCACGATGGCGAGGGCGCCTTCCATGGAGACGCAAAAGCCCTTCAGCGGCCAGTAGCATCTGACTTTGAGCCTTCAGGGTCTGAG

Translation of the sequence ([3'5' Frame 2](http://web.expasy.org/cgi-bin/translate/dna_sequences?/work/expasy/tmp/http/seqdna.8623,6)):

IGQCQLHDGEGAFHGDAKALQRPVASDFEPSGSE

Alignment of the sequence against BCR-ABL (p190) fusion protein sequence:

**88.5% identity in 26 aa overlap (7-32:3-28); score: 165 E(10000): 3.4e-12**

10 20 30

B2 HDGEGAFHGDAKALQRPVASDFEPSG

.::::::::::.::::::::::::.:

BCR-AB NDGEGAFHGDAEALQRPVASDFEPQG

10 20

**II.2b.** # **P144** clone B2

Sequence of the clone (with M13/pUC-F sequencing primer):

CATGCATGATTACGATTCGAGCTCGGTACCCGGGGATCCCTCAGACCCTGAAGGCTCAAAGTCAGATGCTACTGGCCGCTGAAGGGCTTCTGCGTCTCCATGGAAGGCGCCCTCGCCATCGTTGAAGCTTGGCACTGGCCGTCGTTTTACAACGTCGTGACTGGGAAAACCCTGGCGTTACCCAACTTAATCGCCTTGCAGCACATCCCCCTTTCGCCAGCTGGCGTAATAGCGAAGAGGCCCGCACCGATCGCCCTTCCCAACAGTTGCGCAGCCTGAATGGCGAATGGCGCCTGATGCGGTATTTTCTCCTTACGCATCTGTGCGGTATTTCACACCGCATATGGTGCACTCTCAGTACAATCTGCTCTGATGCCGCATAGTTAAGCCAGCCCCGACACCCGCCAACACCCGCTGACGCGCCCTGACGGGCTTGTCTGCTCCCGGCATCCGCTTACAGACAAGCTGTGACCGTCTCCGGGAGCTGCATGTGTCAGAGGTTTTCACCGTCATCACCGAAACGCGCGAGACGAAAGGGCCTCGTGATACGCCTATTTTTATAGGTTAATGTCATGATAATAATGGTTTCTTAGACGTCAGGTGGCACTTTTCGGGGAAATGTGCGCGGAACCCCTATTTGTTTATTTTTCTAAATACATTCAAATATGTATCCGCTCATGAGACAATAACCCTGATAAATGCTTCAATAATATTGAAAAAGGAAGAGTATGAGTATTCAACATTTCCGTGTCGCCCTTATTCCCTTTTTTTGCGGCATTTTGCCTTCCTGTTTTTGCTCACCCAGAAACGCTGGTGAAAGTAAAAGATGCTGAAGATCAGTTGGGTGCACGAGTGGGTTTACATCGAACTGGATCTCAACAGCGGGTAAGATCCCTTGAGAGTTTTCGCCCCCGAAGAACCGTTTTCCAATGATGAGCACTTTTAAGTTCTGCTATGTGCGCGTATATCCCGTATTGACCGCCGGCAAGAGCACTCGTCCGCCCGCATACACTAATCTCAGGATTGACTGGTCAGTACTCCACCCAGTCCACAGAAGCATCTTACCGTATGGCCATGACAGGTTGAAGCAGCAT

Alignment of the sequence against published BCR-ABL (p190) FT sequence:

**97.7% identity** (97.7% similar) in 87 nt overlap (124-38:7-92)

120 110 100 90 80 70

B2 CAACGATGGCGAGGGCGCCTTCCATGGAGACGCAGAAGCCCTTCAGCGGCCAGTAGCATC

::::::::::::::::::::::::::::::::::::::::::::::::::::::::::::

BCR-ABL CAACGATGGCGAGGGCGCCTTCCATGGAGACGCAGAAGCCCTTCAGCGGCCAGTAGCATC

10 20 30 40 50 60

60 50 40

B2 TGACTTTGAGCCTTCAGGGTCTGAGGG

::::::::::::: ::::::::::: :

BCR-ABL TGACTTTGAGCCT-CAGGGTCTGAGTG

70 80 90

Translation of the sequence ([3'5' Frame 3](http://web.expasy.org/cgi-bin/translate/dna_sequences?/work/expasy/tmp/http/seqdna.8623,6)):

AASTCHGHTVRCFCGLGGVLTSQS-D-CMRADECSCRRSIRDIRAHSRT-KCSSLENGSS

GAKTLKGSYPLLRSSSM-THSCTQLIFSIFYFHQRFWVSKNRKAKCRKKRE-GRHGNVEY

SYSSFFNIIEAFIRVIVS-ADTYLNVFRKINK-GFRAHFPEKCHLTSKKPLLS-H-PIKI

GVSRGPFVSRVSVMTVKTSDTCSSRRRSQLVCKRMPGADKPVRARQRVLAGVGAGLTMRH

QSRLY-ECTICGVKYRTDA-GENTASGAIRHSGCATVGKGDRCGPLRYYASWRKGDVLQG

D-VG-RQGFPSHDVVKRRPVPSFNDGEGAFHGDAEALQRPVASDFEPSGSEGSPGTELES

-SCM

Alignment of the sequence against BCR-ABL (p190) fusion protein sequence:

**96.2% identity** (100.0% similar) in 26 aa overlap (311-336:3-28)

320 330

B2 NDGEGAFHGDAEALQRPVASDFEPSG

::::::::::::::::::::::::.:

BCR-ABL NDGEGAFHGDAEALQRPVASDFEPQG

10 20

**II.3a.** # **P145** clone B3

Sequence of the clone (with M13/pUC-R sequencing primer):

CCTGCGGTCAGTGCAGCTTCACGATGGCGAGGGCGCCTTCCATGGAGACGCAAAAGCCCTTCAGCGGCCAGTAGCATCTGACTTTGAGCCTTCAGGGTCTGAGGGATCCCCGGGTACCGAGCTCGAATTCGTAATCATGTCATAGCTGTTTCCTGTGTGAAATTGTTATCCGCTCACAATTCCACACAACATACGAGCCGGAAGCATAAAGTGTAAAGCCTGGGGTGCCTAATGAGTGAGCTAACTCACATTAATTGCGTTGCGCTCACTGCCCGCTTTCCAGTCGGGAAACCTGTCGTGCCAGCTGCATTAATGAATCGGCCAACGCGCGGGGAGAGGCGGTTTGCGTATTGGGCGCTCTTCCGCTTCCTCGCTCACTGACTCGCTGCGCTCGGTCGTTCGGCTGCGGCGAGCGGTATCAGCTCACTCAAAGGCGGTAATACGGTTATCCACAGAATCAGGGGATAACGCAGGAAAGAACATGTGAGCAAAAGGCCAGCAAAAGGCCAGGAACCGTAAAAAGGCCGCGTTGCTGGCGTTTTTCCATAGGCTCCGCCCCCCTGACGAGCATCACAAAAATCGACGCTCAAGTCAGAGGTGGCGAAACCCGACAGGACTATAAAGATACCAGGCGTTTCCCCCTGGAAGCTCCCTCGTGCGCTCTCCTGTTCCGACCCTGCCGCTTACCGGATACCTGTCCGCCTTTCTCCCTTCGGGAAGCGTGGCGCTTTCTCATAGCTCACGCTGTAGGTATCTCAGTTCGGTGTAGGTCGTTCGCTCCAAGCTGGGCTGTGTGCACGAACCCCCCCGTTCAGCCCGACCGCTGCGCCTTATCCGGTAACTATCGTCTTGAGTCCAACCCGGTAAGACACGACTTATCGCCACTGGCAGCAGCCCACTGGTAACAGGGATTAGCAGAGCGAGGTATGTAGGCGGTGCTACAGAGTTCTTGGAAGTGGGTGGCCCTACTACCGGCCTACACTAGAAGAACAGTATTTGGGTATCTGCGCCTCTGCTGAAGCCCAGCTAACCCTTCGGAAAAGAGTTGGTAAGCTCGTGATCCGGCCAAACAACCACCGCTTGGTAGCCGATGATTTTTTGCTTTGCCAAGGTCAAGCCCAG

Alignment of the sequence against published BCR-ABL (p190) FT sequence:

**96.5% identity** (96.5% similar) in 85 nt overlap (21-105:9-92)

30 40 50 60 70 80

B3 ACGATGGCGAGGGCGCCTTCCATGGAGACGCAAAAGCCCTTCAGCGGCCAGTAGCATCTG

:::::::::::::::::::::::::::::::: :::::::::::::::::::::::::::

BCR-ABL ACGATGGCGAGGGCGCCTTCCATGGAGACGCAGAAGCCCTTCAGCGGCCAGTAGCATCTG

10 20 30 40 50 60

90 100

B3 ACTTTGAGCCTTCAGGGTCTGAGGG

::::::::::: ::::::::::: :

BCR-ABL ACTTTGAGCCT-CAGGGTCTGAGTG

70 80 90

Sequence from START to BamHI-cloning site:

CCTGCGGTCAGTGCAGCTTCACGATGGCGAGGGCGCCTTCCATGGAGACGCAAAAGCCCTTCAGCGGCCAGTAGCATCTGACTTTGAGCCTTCAGGGTCTGAG

Translation of the sequence ([3'5' Frame 2](http://web.expasy.org/cgi-bin/translate/dna_sequences?/work/expasy/tmp/http/seqdna.8623,6)):

LRSVQLHDGEGAFHGDAKALQRPVASDFEPSGSE

Alignment of the sequence against BCR-ABL (p190) fusion protein sequence:

**88.5% identity in 26 aa overlap** (7-32:3-28); score: 165 E(10000): 3.4e-12

10 20 30

B3 HDGEGAFHGDAKALQRPVASDFEPSG

.::::::::::.::::::::::::.:

BCR-AB NDGEGAFHGDAEALQRPVASDFEPQG

10 20

**II.3b.** # **P145** clone B3

Sequence of the clone (with M13/pUC-F sequencing primer):

CAGGGATGATTACGATTCGAGCTCGGTACCCGGGGATCCCTCAGACCCTGAAGGCTCAAAGTCAGATGCTACTGGCCGCTGAAGGGCTTCTGCGTCTCCATGGAAGGCGCCCTCGCCATCGTTGAAGCTTGGCACTGGCCGTCGTTTTACAACGTCGTGACTGGGAAAACCCTGGCGTTACCCAACTTAATCGCCTTGCAGCACATCCCCCTTTCGCCAGCTGGCGTAATAGCGAAGAGGCCCGCACCGATCGCCCTTCCCAACAGTTGCGCAGCCTGAATGGCGAATGGCGCCTGATGCGGTATTTTCTCCTTACGCATCTGTGCGGTATTTCACACCGCATATGGTGCACTCTCAGTACAATCTGCTCTGATGCCGCATAGTTAAGCCAGCCCCGACACCCGCCAACACCCGCTGACGCGCCCTGACGGGCTTGTCTGCTCCCGGCATCCGCTTACAGACAAGCTGTGACCGTCTCCGGGAGCTGCATGTGTCAGAGGTTTTCACCGTCATCACCGAAACGCGCGAGACGAAAGGGCCTCGTGATACGCCTATTTTTATAGGTTAATGTCATGATAATAATGGTTTCTTAGACGTCAGGTGGCACTTTTCGGGGAAATGTGCGCGGAACCCCTATTTGTTTATTTTTCTAAATACATTCAAATATGTATCCGCTCATGAGACAATAACCCTGATAAATGCTTCAATAATATTGAAAAAGGAAGAGTATGAGTATTCAACATTTCCGTGTCGCCCTTATTCCCTTTTTTTGCGGCATTTTGCCTTCCTGTTTTTGCTCACCCAGAAACGCTGGTGAAAGTAAAAGATGCTGAAGATCAGTTGGGTGCACGAGTGGGTTACATCGAACTGGGATCTCAACAGCGGTAAGATCCCTTGAGAGTTTTCGCCCCCGAAGAACGTTTTCCAATGATGAGCACTTTTAAAGTTCTGCTATGTGGGCGCGGTATTATCCCGTATTGACGCGGCAGAGCACTCGTCGCGCATAACACTTATCTCAGATGACTTGTTGAGTACTTCACAAGTCACAGGAAAAGGCACTATACGATGACATGACTGTTAGAGACTTATCGCAGTGCTG

Alignment of the sequence against published BCR-ABL (p190) FT sequence:

**97.7% identity** (97.7% similar) in 87 nt overlap (124-38:7-92)

120 110 100 90 80 70

B3 CAACGATGGCGAGGGCGCCTTCCATGGAGACGCAGAAGCCCTTCAGCGGCCAGTAGCATC

::::::::::::::::::::::::::::::::::::::::::::::::::::::::::::

BCR-ABL CAACGATGGCGAGGGCGCCTTCCATGGAGACGCAGAAGCCCTTCAGCGGCCAGTAGCATC

10 20 30 40 50 60

60 50 40

B3 TGACTTTGAGCCTTCAGGGTCTGAGGG

::::::::::::: ::::::::::: :

BCR-ABL TGACTTTGAGCCT-CAGGGTCTGAGTG

70 80 90

Translation of the sequence ([3'5' Frame 2](http://web.expasy.org/cgi-bin/translate/dna_sequences?/work/expasy/tmp/http/seqdna.8623,6)):

STAISL-QSCHRIVPFPVTCEVLNKSSEISVMRDECSAASIRDNTAPT-QNFKSAHHWKT

FFGGENSQGILPLLRSQFDVTHSCTQLIFSIFYFHQRFWVSKNRKAKCRKKRE-GRHGNV

EYSYSSFFNIIEAFIRVIVS-ADTYLNVFRKINK-GFRAHFPEKCHLTSKKPLLS-H-PI

KIGVSRGPFVSRVSVMTVKTSDTCSSRRRSQLVCKRMPGADKPVRARQRVLAGVGAGLTM

RHQSRLY-ECTICGVKYRTDA-GENTASGAIRHSGCATVGKGDRCGPLRYYASWRKGDVL

QGD-VG-RQGFPSHDVVKRRPVPSFNDGEGAFHGDAEALQRPVASDFEPSGSEGSPGTEL

ES-SSL

Alignment of the sequence against BCR-ABL (p190) fusion protein sequence:

**96.2% identity** (100.0% similar) in 26 aa overlap (315-340:3-28)

320 330 340

B3 NDGEGAFHGDAEALQRPVASDFEPSG

::::::::::::::::::::::::.:

BCR-ABL NDGEGAFHGDAEALQRPVASDFEPQG

10 20

**II.4a.** # **P217** clone B4

Sequence of the clone (with M13/pUC-R sequencing primer):

CCTCAGGCCGTGCCAGCTTCACGATGGCGAGGGCGCCTTCCATGGAGACGCAAAAGCCCTTCAGCGGCCAGTAGCATCTGACTTTGAGCCTTCAGGGTCTGAGGGATCCCCGGGTACCGAGCTCGAATTCGTAATCATGTCATAGCTGTTTCCTGTGTGAAATTGTTATCCGCTCACAATTCCACACAACATACGAGCCGGAAGCATAAAGTGTAAAGCCTGGGGTGCCTAATGAGTGAGCTAACTCACATTAATTGCGTTGCGCTCACTGCCCGCTTTCCAGTCGGGAAACCTGTCGGGCCAGCTGCATTAATGAATCGGCCAACGCGCGGGGAGAGGCGGTTTGCGTATTGGGCGCTCTTCCGCTTCCTCGCTCACTGACTCGCTGCGCTCGGTCGTTCGGCTGCGGCGAGCGGTATCAGCTCACTCAAAGGCGGTAATACGGTTATCCACAGAATCAGGGGATAACGCAGGAAAGAACATGTGAGCAAAAGGCCAGCAAAAGGCCAGGAACCGTAAAAAGGCCGCGTTGCTGGCGTTTTTCCATAGGCTCCGCCCCCCTGACGAGCATCACAAAAATCGACGCTCAAGTCAGAGGTGGCGAAACCCGACAGGACTATAAAGATACCAGGCGTTTCCCCCTGGAAGCTCCCTCGTGCGCTCTCCTGTTCCGACCCTGCCGCTTACCGGATACCTGTCCGCCTTTCTCCCTTCGGGAAGCGTGGCGCTTTCTCATAGCTCACGCTGTAGGTATCTCAGTTCGGTGTAGGTCGTTCGCTCCAAGCTGGGCTGTGTGCACGAACCCCCCGTTCAGCCCGACCGCTGCGCCTTATCCGGTAACTATCGTCTTGAGTCCAACCCGGTAAGACACGACTTATCGCCCACTGGCAGCAGCCCACTGGTAACAGGATTAGCAGAGCGAGGTATGTAGCCGGTGCTACAGAGTTCTTGAAGTGGTGCCTAACTACGGCTACACTAGAAAGAACAGGTATTGTATCTGCGCTCTGCTGGAGCAAGCTACGTCGAAAAGAGTGGTAGCTCTGATCGCCAACAACACGCTGTACCGATGCTTTTGGTTGGCCAGGCAGAC

Alignment of the sequence against published BCR-ABL (p190) FT sequence:

**96.5% identity** (96.5% similar) in 85 nt overlap (21-105:9-92)

30 40 50 60 70 80

B4 ACGATGGCGAGGGCGCCTTCCATGGAGACGCAAAAGCCCTTCAGCGGCCAGTAGCATCTG

:::::::::::::::::::::::::::::::: :::::::::::::::::::::::::::

BCR-ABL ACGATGGCGAGGGCGCCTTCCATGGAGACGCAGAAGCCCTTCAGCGGCCAGTAGCATCTG

10 20 30 40 50 60

90 100

B4 ACTTTGAGCCTTCAGGGTCTGAGGG

::::::::::: ::::::::::: :

BCR-ABL ACTTTGAGCCT-CAGGGTCTGAGTG

70 80 90

Sequence from START to BamHI-cloning site:

CCTCAGGCCGTGCCAGCTTCACGATGGCGAGGGCGCCTTCCATGGAGACGCAAAAGCCCTTCAGCGGCCAGTAGCATCTGACTTTGAGCCTTCAGGGTCTGAG

Translation of the sequence ([3'5' Frame 2](http://web.expasy.org/cgi-bin/translate/dna_sequences?/work/expasy/tmp/http/seqdna.8623,6)):

LRPCQLHDGEGAFHGDAKALQRPVASDFEPSGSE

Alignment of the sequence against BCR-ABL (p190) fusion protein sequence:

**88.5% identity in 26 aa overlap (7-32:3-28); score: 165 E(10000): 3.4e-12**

10 20 30

B4 HDGEGAFHGDAKALQRPVASDFEPSG

.::::::::::.::::::::::::.:

BCR-ABL NDGEGAFHGDAEALQRPVASDFEPQG

10 20

**II.4b.** # **P217** clone B4

Sequence of the clone (with M13/pUC-F sequencing primer):

CATCATGATTACGATTCGAGCTCGGTACCCGGGGATCCCTCAGACCCTGAAGGCTCAAAGTCAGATGCTACTGGCCGCTGAAGGGCTTCTGCGTCTCCATGGAAGGCGCCCTCGCCATCGTTGAAGCTTGGCACTGGCCGTCGTTTTACAACGTCGTGACTGGGAAAACCCTGGCGTTACCCAACTTAATCGCCTTGCAGCACATCCCCCTTTCGCCAGCTGGCGTAATAGCGAAGAGGCCCGCACCGATCGCCCTTCCCAACAGTTGCGCAGCCTGAATGGCGAATGGCGCCTGATGCGGTATTTTCTCCTTACGCATCTGTGCGGTATTTCACACCGCATATGGTGCACTCTCAGTACAATCTGCTCTGATGCCGCATAGTTAAGCCAGCCCCGACACCCGCCAACACCCGCTGACGCGCCCTGACGGGCTTGTCTGCTCCCGGCATCCGCTTACAGACAAGCTGTGACCGTCTCCGGGAGCTGCATGTGTCAGAGGTTTTCACCGTCATCACCGAAACGCGCGAGACGAAAGGGCCTCGTGATACGCCTATTTTTATAGGTTAATGTCATGATAATAATGGTTTCTTAGACGTCAGGTGGCACTTTTCGGGGAAATGTGCGCGGAACCCCTATTTGTTTATTTTTCTAAATACATTCAAATATGTATCCGCTCATGAGACAATAACCCTGATAAATGCTTCAATAATATTGAAAAAGGAAGAGTATGAGTATTCAACATTTCCGTGTCGCCCTTATTCCCTTTTTTGCGGCATTTTGCCTTCCTGTTTTTGCTCACCCAGAAACGCTGGTGAAAGTAAAAGATGCTGAAGATCAGTTGGGTGCACGAGTGGGTTACATCGAACTGGATCTCAACAGCGGTAAGATCCTTGAGAGTTTTCGCCCCGAAGACGTTTTCCAATGATGAGCACTTTTAAGTCTGCTATGTGGCGCGTATATCCCCGTATGACGCGGCAGAGCCACTCGTTCGGCGCATAACACTATCTCAGATGACTTGTTGAGTACTCACAGTCACAAGAAAGCCATCCTACCGGTATGCATGAACTGTAGAGATTATGCATGGCTGACCATAAGCCTGATG

Alignment of the sequence against published BCR-ABL (p190) FT sequence:

**97.7% identity** (97.7% similar) in 87 nt overlap (123-37:7-92)

120 110 100 90 80 70

B4 CAACGATGGCGAGGGCGCCTTCCATGGAGACGCAGAAGCCCTTCAGCGGCCAGTAGCATC

::::::::::::::::::::::::::::::::::::::::::::::::::::::::::::

BCR-ABL CAACGATGGCGAGGGCGCCTTCCATGGAGACGCAGAAGCCCTTCAGCGGCCAGTAGCATC

10 20 30 40 50 60

60 50 40

B4 TGACTTTGAGCCTTCAGGGTCTGAGGG

::::::::::::: ::::::::::: :

BCR-ABL TGACTTTGAGCCT-CAGGGTCTGAGTG

70 80 90

Translation of the sequence ([3'5' Frame 3](http://web.expasy.org/cgi-bin/translate/dna_sequences?/work/expasy/tmp/http/seqdna.8623,6)):

SGLWSAMHNLYSSCIPVGWLSCDCEYSTSHLR-CYAPNEWLCRVIRGYTRHIADLKVLII

GKRLRGENSQGSYRC-DPVRCNPLVHPTDLQHLLLSPAFLGEQKQEGKMPQKRE-GRHGN

VEYSYSSFFNIIEAFIRVIVS-ADTYLNVFRKINK-GFRAHFPEKCHLTSKKPLLS-H-P

IKIGVSRGPFVSRVSVMTVKTSDTCSSRRRSQLVCKRMPGADKPVRARQRVLAGVGAGLT

MRHQSRLY-ECTICGVKYRTDA-GENTASGAIRHSGCATVGKGDRCGPLRYYASWRKGDV

LQGD-VG-RQGFPSHDVVKRRPVPSFNDGEGAFHGDAEALQRPVASDFEPSGSEGSPGTE

LES-S-

Alignment of the sequence against BCR-ABL (p190) fusion protein sequence:

**96.2% identity** (100.0% similar) in 26 aa overlap (316-341:3-28)

320 330 340

B4 NDGEGAFHGDAEALQRPVASDFEPSG

::::::::::::::::::::::::.:

BCR-ABL NDGEGAFHGDAEALQRPVASDFEPQG

10 20

**II.5a.** # **P233** clone B5

Sequence of the clone (with M13/pUC-R sequencing primer):

CATCCGCCAGGTGCCAGCTTCACGATGGCGAGGGCGCCTTCCATGGAGACGCAAAAGCCCTTCAGCGGCCAGTAGCATCTGACTTTGAGCCTTCAGGGTCTGAGGGATCCCCGGGTACCGAGCTCGAATTCGTAATCATGTCATAGCTGTTTCCTGTGTGAAATTGTTATCCGCTCACAATTCCACACAACATACGAGCCGGAAGCATAAAGTGTAAAGCCTGGGGTGCCTAATGAGTGAGCTAACTCACATTAATTGCGTTGCGCTCACTGCCCGCTTTCCAGTCGGGAAACCTGTCGTGCCAGCTGCATTAATGAATCGGCCAACGCGCGGGGAGAGGCGGTTTGCGTATTGGGCGCTCTTCCGCTTCCTCGCTCACTGACTCGCTGCGCTCGGTCGTTCGGCTGCGGCGAGCGGTATCAGCTCACTCAAAGGCGGTAATACGGTTATCCACAGAATCAGGGGATAACGCAGGAAAGAACATGTGAGCAAAAGGCCAGCAAAAGGCCAGGAACCGTAAAAAGGCCGCGTTGCTGGCGTTTTTCCATAGGCTCCGCCCCCCTGACGAGCATCACAAAAATCGACGCTCAAGTCAGAGGTGGCGAAACCCGACAGGACTATAAAGATACCAGGCGTTTCCCCCTGGAAGCTCCCTCGTGCGCTCTCCTGTTCCGACCCTGCCGCTTACCGGATACCTGTCCGCCTTTCTCCCTTCGGGAAGCGTGGCGCTTTCTCATAGCTCACGCTGTAGGTATCTCAGTTCGGTGTAGGTCGTTCGCTCCAAGCTGGGCTGTGTGCACGAAACCCCCCGTTCAGCCCGACCGCTGCGCCCTTATCCGGTAACTATCGTCTTGAGTCCAACCCGGTAAGACACGACTTATCGCCCACTGGCAGCAGCCACTGGTAACAGGATTAGCAGAGCGAGGTATGTAGGCGGTGCTACAGAGTTCTTGAAGTGGTGCCTACTACGGGCTACACTAGAAGAACAGTATTTGGTATCTGCGCTCTGCTGGAAGCCAGTACTTCGGAAAAAGAGTGCTAGCTCGTGATTCGGCAACAAACACCGCTGGCTAGCGATGCGTTTTGTCGCAAGGCCAGCAGAACTAG

Alignment of the sequence against published BCR-ABL (p190) FT sequence:

**96.5% identity** (96.5% similar) in 85 nt overlap (22-106:9-92)

30 40 50 60 70 80

B5 ACGATGGCGAGGGCGCCTTCCATGGAGACGCAAAAGCCCTTCAGCGGCCAGTAGCATCTG

:::::::::::::::::::::::::::::::: :::::::::::::::::::::::::::

BCR-ABL ACGATGGCGAGGGCGCCTTCCATGGAGACGCAGAAGCCCTTCAGCGGCCAGTAGCATCTG

10 20 30 40 50 60

90 100

B5 ACTTTGAGCCTTCAGGGTCTGAGGG

::::::::::: ::::::::::: :

BCR-ABL ACTTTGAGCCT-CAGGGTCTGAGTG

70 80 90

Sequence from START to BamHI-cloning site:

CATCCGCCAGGTGCCAGCTTCACGATGGCGAGGGCGCCTTCCATGGAGACGCAAAAGCCCTTCAGCGGCCAGTAGCATCTGACTTTGAGCCTTCAGGGTCTGAG

Translation of the sequence ([3'5' Frame 3](http://web.expasy.org/cgi-bin/translate/dna_sequences?/work/expasy/tmp/http/seqdna.8623,6)):

SARCQLHDGEGAFHGDAKALQRPVASDFEPSGSE

**88.5% identity in 26 aa overlap** (7-32:3-28); score: 165 E(10000): 3.4e-12

10 20 30

B5 HDGEGAFHGDAKALQRPVASDFEPSG

.::::::::::.::::::::::::.:

BCR-ABL NDGEGAFHGDAEALQRPVASDFEPQG

10 20

**II.5b.** # **P233** clone B5

Sequence of the clone (with M13/pUC-F sequencing primer):

CATTCATCATTACGATTCGAGCTCGGTACCCGGGGATCCCTCAGACCCTGAAGGCTCAAAGTCAGATGCTACTGGCCGCTGAAGGGCTTCTGCGTCTCCATGGAAGGCGCCCTCGCCATCGTTGAAGCTTGGCACTGGCCGTCGTTTTACAACGTCGTGACTGGGAAAACCCTGGCGTTA

CCCAACTTAATCGCCTTGCAGCACATCCCCCTTTCGCCAGCTGGCGTAATAGCGAAGAGGCCCGCACCGATCGCCCTTCCCAACAGTTGCGCAGCCTGAATGGCGAATGGCGCCTGATGCGGTATTTTCTCCTTACGCATCTGTGCGGTATTTCACACCGCATATGGTGCACTCTCAGTA

CAATCTGCTCTGATGCCGCATAGTTAAGCCAGCCCCGACACCCGCCAACACCCGCTGACGCGCCCTGACGGGCTTGTCTGCTCCCGGCATCCGCTTACAGACAAGCTGTGACCGTCTCCGGGAGCTGCATGTGTCAGAGGTTTTCACCGTCATCACCGAAACGCGCGAGACGAAAGGGCC

TCGTGATACGCCTATTTTTATAGGTTAATGTCATGATAATAATGGTTTCTTAGACGTCAGGTGGCACTTTTCGGGGAAATGTGCGCGGAACCCCTATTTGTTTATTTTTCTAAATACATTCAAATATGTATCCGCTCATGAGACAATAACCCTGATAAATGCTTCAATAATATTGAAAAA

GGAAGAGTATGAGTATTCACATTTCCGTGTCGCCCTTATTCCCCTTTTTTGCGGCATTTTGCCTTCCTGTTTTTTGCTCCACCCAGAACGCTGTGAAAGTAAAAGATGCTGAAGATCAGGTGGTGCACGAGTGGTTACATCGAACTGATCTCACAGCGGTAGGATCTGAGAAGTTTCGCT

CGAGACGGTTTCAATGAATGACACTTTAAGTTCTGGCTATGTGGCCGGATTATCCGAATTGACCGCAGAGCACTCGCTCGCCGCTAACATTACCGATGACTGGTGATATCTACA

Alignment of the sequence against published BCR-ABL (p190) FT sequence:

**97.7% identity** (97.7% similar) in 87 nt overlap (124-38:7-92)

120 110 100 90 80 70

B5 CAACGATGGCGAGGGCGCCTTCCATGGAGACGCAGAAGCCCTTCAGCGGCCAGTAGCATC

::::::::::::::::::::::::::::::::::::::::::::::::::::::::::::

BCR-ABL CAACGATGGCGAGGGCGCCTTCCATGGAGACGCAGAAGCCCTTCAGCGGCCAGTAGCATC

10 20 30 40 50 60

60 50 40

B5 TGACTTTGAGCCTTCAGGGTCTGAGGG

::::::::::::: ::::::::::: :

BCR-ABL TGACTTTGAGCCT-CAGGGTCTGAGTG

70 80 90

Translation of the sequence ([3'5' Frame 1](http://web.expasy.org/cgi-bin/translate/dna_sequences?/work/expasy/tmp/http/seqdna.8623,6)):

CRYHQSSVMLAASECSAVNSDNPAT-PELKVSFIETVSSETSQILPL-DQFDVTTRAPPD

LQHLLLSQRSGWSKKQEGKMPQKRGIRATRKCEYSYSSFFNIIEAFIRVIVS-ADTYLNV

FRKINK-GFRAHFPEKCHLTSKKPLLS-H-PIKIGVSRGPFVSRVSVMTVKTSDTCSSRR

RSQLVCKRMPGADKPVRARQRVLAGVGAGLTMRHQSRLY-ECTICGVKYRTDA-GENTAS

GAIRHSGCATVGKGDRCGPLRYYASWRKGDVLQGD-VG-RQGFPSHDVVKRRPVPSFNDG

EGAFHGDAEALQRPVASDFEPSGSEGSPGTELES---M

Alignment of the sequence against BCR-ABL (p190) fusion protein sequence:

**96.2% identity** (100.0% similar) in 26 aa overlap (288-313:3-28)

290 300 310

B5 NDGEGAFHGDAEALQRPVASDFEPSG

::::::::::::::::::::::::.:

BCR-ABL NDGEGAFHGDAEALQRPVASDFEPQG

10 20

**II.6a.** # **P522** clone B6

Sequence of the clone (with M13/pUC-R sequencing primer):

CATCCGGCAGTGCCAGCTTCACGATGGCGAGGGCGCCTTCCATGGAGACGCAAAAGCCCTTCAGCGGCCAGTAGCATCTGACTTTGAGCCTTCAGGGTCTGAGGGATCCCCGGGTACCGAGCTCGAATTCGTAATCATGTCATAGCTGTTTCCTGTGTGAAATTGTTATCCGCTCACAATTCCACACAACATACGAGCCGGAAGCATAAAGTGTAAAGCCTGGGGTGCCTAATGAGTGAGCTAACTCACATTAATTGCGTTGCGCTCACTGCCCGCTTTCCAGTCGGGAAACCTGTCGTGCCAGCTGCATTAATGAATCGGCCAACGCGCGGGGAGAGGCGGTTTGCGTATTGGGCGCTCTTCCGCTTCCTCGCTCACTGACTCGCTGCGCTCGGTCGTTCGGCTGCGGCGAGCGGTATCAGCTCACTCAAAGGCGGTAATACGGTTATCCACAGAATCAGGGGATAACGCAGGAAAGAACATGTGAGCAAAAGGCCAGCAAAAGGCCAGGAACCGTAAAAAGGCCGCGTTGCTGGCGTTTTTCCATAGGCTCCGCCCCCCTGACGAGCATCACAAAAATCGACGCTCAAGTCAGAGGTGGCGAAACCCGACAGGACTATAAAGATACCAGGCGTTTCCCCCTGGAAGCTCCCTCGTGCGCTCTCCTGTTCCGACCCTGCCGCTTACCGGATACCTGTCCGCCTTTCTCCCTTCGGGAAGCGTGGCGCTTTTCTCATAGCTCACGCTGTAGGTATCTCAGTTCGGTGTAGGTCGTTCGCTCCAAGCTGGGCTGTGTGCACGAACCCCCCGTTCAGCCCGGACCGCTGCGCCTTATCCGGTAACTATCGTCTTGAGTCCAACCCGGGTAAGACACGACTTATCGCCCACTTGCCAGCAGCCACTGTAACAGGATTAGCAGAGGCGAGGTATGTAGGCGGTGCTACAGAGTTCTTGAGTGTGCCCTACTACGGCCTACCTAGAGACAGTATTTGTATCTGCGCTCTTGCTGAGCCAGTAACGTCCGGAAAGAGTTGTAGCTCTGGATTCAGCCGACACACGCTGGTAACGTGCTTTTGGTTGC

Alignment of the sequence against published BCR-ABL (p190) FT sequence:

**96.5% identity** (96.5% similar) in 85 nt overlap (21-105:9-92)

30 40 50 60 70 80

B6 ACGATGGCGAGGGCGCCTTCCATGGAGACGCAAAAGCCCTTCAGCGGCCAGTAGCATCTG

:::::::::::::::::::::::::::::::: :::::::::::::::::::::::::::

BCR-ABL ACGATGGCGAGGGCGCCTTCCATGGAGACGCAGAAGCCCTTCAGCGGCCAGTAGCATCTG

10 20 30 40 50 60

90 100

B6 ACTTTGAGCCTTCAGGGTCTGAGGG

::::::::::: ::::::::::: :

BCR-ABL ACTTTGAGCCT-CAGGGTCTGAGTG

70 80 90

Sequence from START to BamHI-cloning site:

CATCCGGCAGTGCCAGCTTCACGATGGCGAGGGCGCCTTCCATGGAGACGCAAAAGCCCTTCAGCGGCCAGTAGCATCTGACTTTGAGCCTTCAGGGTCTGAG

Translation of the sequence ([3'5' Frame 2](http://web.expasy.org/cgi-bin/translate/dna_sequences?/work/expasy/tmp/http/seqdna.8623,6)):

IRQCQLHDGEGAFHGDAKALQRPVASDFEPSGSE

Alignment of the sequence against BCR-ABL (p190) fusion protein sequence:

10 20 30

B6 HDGEGAFHGDAKALQRPVASDFEPSG

.::::::::::.::::::::::::.:

BCR-AB NDGEGAFHGDAEALQRPVASDFEPQG

10 20

**II.6b.** # **P522** clone B6

Sequence of the clone (with M13/pUC-F sequencing primer):

CATTCATGCATTACGATTCGAGCTCGGTACCCGGGGATCCCTCAGACCCTGAAGGCTCAAAGTCAGATGCTACTGGCCGCTGAAGGGCTTCTGCGTCTCCATGGAAGGCGCCCTCGCCATCGTTGAAGCTTGGCACTGGCCGTCGTTTTACAACGTCGTGACTGGGAAAACCCTGGCGTT

ACCCAACTTAATCGCCTTGCAGCACATCCCCCTTTCGCCAGCTGGCGTAATAGCGAAGAGGCCCGCACCGATCGCCCTTCCCAACAGTTGCGCAGCCTGAATGGCGAATGGCGCCTGATGCGGTATTTTCTCCTTACGCATCTGTGCGGTATTTCACACCGCATATGGTGCACTCTCAGT

ACAATCTGCTCTGATGCCGCATAGTTAAGCCAGCCCCGACACCCGCCAACACCCGCTGACGCGCCCTGACGGGCTTGTCTGCTCCCGGCATCCGCTTACAGACAAGCTGTGACCGTCTCCGGGAGCTGCATGTGTCAGAGGTTTTCACCGTCATCACCGAAACGCGCGAGACGAAAGGGC

CTCGTGATACGCCTATTTTTATAGGTTAATGTCATGATAATAATGGTTTCTTAGACGTCAGGTGGCACTTTTCGGGGAAATGTGCGCGGAACCCCTATTTGTTTATTTTTCTAAATACATTCAAATATGTATCCGCTCATGAGACATAACCCTGATAAATGCTTCATATATTGAAAAAGG

AAGAGTATGAGTATTCACATTCCGTGTCGCCCTTATTCCCTTTTTGCGCATTTTGCCTTCCTGTTTTTGCCTCCACCCAGAAACGCTGTGAAAGTAAAAGATGCTGAAGATCAGGTGGGTGCCACGAGTGGTACATCGACTGATCTCAACAGCGTAGATCTTGAAGTTTCGCCTCGAGAC

GGTTCATGATGAGCACTTAAGGTCTGCTATTGGGCGCGGATATCTGGATGACCGCAAGCACTCGATCGCGCAGTACCATTTCTCAGAATGACTTGG

Alignment of the sequence against published BCR-ABL (p190) FT sequence:

**97.7% identity** (97.7% similar) in 87 nt overlap (125-39:7-92)

120 110 100 90 80 70

B6 CAACGATGGCGAGGGCGCCTTCCATGGAGACGCAGAAGCCCTTCAGCGGCCAGTAGCATC

::::::::::::::::::::::::::::::::::::::::::::::::::::::::::::

BCR-ABL CAACGATGGCGAGGGCGCCTTCCATGGAGACGCAGAAGCCCTTCAGCGGCCAGTAGCATC

10 20 30 40 50 60

60 50 40

B6 TGACTTTGAGCCTTCAGGGTCTGAGGG

::::::::::::: ::::::::::: :

BCR-ABL TGACTTTGAGCCT-CAGGGTCTGAGTG

70 80 90

Translation of the sequence ([3'5' Frame 3](http://web.expasy.org/cgi-bin/translate/dna_sequences?/work/expasy/tmp/http/seqdna.8623,6)):

KSF-EMVLRDRVLAVIQISAPNSRP-VLIMNRLEAKLQDLRC-DQSMYHSWHPPDLQHLL

LSQRFWVEAKTGRQNAQKGNKGDTECEYSYSSFFNI-SIYQGYVS-ADTYLNVFRKINK-

GFRAHFPEKCHLTSKKPLLS-H-PIKIGVSRGPFVSRVSVMTVKTSDTCSSRRRSQLVCK

RMPGADKPVRARQRVLAGVGAGLTMRHQSRLY-ECTICGVKYRTDA-GENTASGAIRHSG

CATVGKGDRCGPLRYYASWRKGDVLQGD-VG-RQGFPSHDVVKRRPVPSFNDGEGAFHGD

AEALQRPVASDFEPSGSEGSPGTELES-CMN

Alignment of the sequence against BCR-ABL (p190) fusion protein sequence:

**96.2% identity** (100.0% similar) in 26 aa overlap (279-304:3-28)

280 290 300

B6 NDGEGAFHGDAEALQRPVASDFEPSG

::::::::::::::::::::::::.:

BCR-ABL NDGEGAFHGDAEALQRPVASDFEPQG

10 20

**II.7.** # **P546** clone B7

Sequence of the clone (with M13/pUC-R sequencing primer):

CATGCATGATTACGATTCGAGCTCGGTACCCGGGGATCCCTCAGACCCTGAAGGCTCAAAGTCAGATGCTACTGGCCGCTGAAGGGCTTCTGCGTCTCCATGGAAGGCGCCCTCGCCATCGTTGAAGCTTGGCACTGGCCGTCGTTTTACAACGTCGTGACTGGGAAAACCCTGGCGTTACCCAACTTAATCGCCTTGCAGCACATCCCCCTTTCGCCAGCTGGCGTAATAGCGAAGAGGCCCGCACCGATCGCCCTTCCCAACAGTTGCGCAGCCTGAATGGCGAATGGCGCCTGATGCGGTATTTTCTCCTTACGCATCTGTGCGGTATTTCACACCGCATATGGTGCACTCTCAGTACAATCTGCTCTGATGCCGCATAGTTAAGCCAGCCCCGACACCCGCCAACACCCGCTGACGCGCCCTGACGGGCTTGTCTGCTCCCGGCATCCGCTTACAGACAAGCTGTGACCGTCTCCGGGAGCTGCATGTGTCAGAGGTTTTCACCGTCATCACCGAAACGCGCGAGACGAAAGGGCCTCGTGATACGCCTATTTTTATAGGTTAATGTCATGATAATAATGGTTTCTTAGACGTCAGGTGGCACTTTTCGGGGAAATGTGCGCGGAACCCCTATTTGTTTATTTTTCTAAATACATTCAAATATGTATCCGCTCATGAGACAATAACCCTGATAAATGCTTCAATAATATTGAAAAAGGAAGAGTATGAGTATTCAACATTTCCGTGTCGCCCTTATTCCCTTTTTTGCGGCATTTTGCCTTCCTGTTTTTGCTCACCCAGAAACGCTGGTGAAAGTAAAAGATGCTGAAGATCAGTTGGGTGCACGAGTGGGTTACATCGAACTGGATCTCAACAGCGGTAAGATCCTTGAGAGTTTTCGCCCCGAAGAACGTTTTCCAATGATGAGCACTTTTAAAGTTCTGCTATGTGGCGCGGTATTATCCCGTATTGACGCCCGGGCAAGAGCAACTCGGTCGCCGCATACACTATTCTCAGAATGACTTTGGTTGAGTACTCACCAGTCACAGAAAAGCATCTTACGGGAATGCAATGACAGTTAGAGACTTATGGCATGCTGGCATTAGCATGAAGTGATAACACTGGCGGACCAACTTAAC

Alignment of the sequence against published BCR-ABL (p190) FT sequence:

**97.7% identity** (97.7% similar) in 87 nt overlap (125-39:7-92)

120 110 100 90 80 70

B7 CAACGATGGCGAGGGCGCCTTCCATGGAGACGCAGAAGCCCTTCAGCGGCCAGTAGCATC

::::::::::::::::::::::::::::::::::::::::::::::::::::::::::::

BCR-ABL CAACGATGGCGAGGGCGCCTTCCATGGAGACGCAGAAGCCCTTCAGCGGCCAGTAGCATC

10 20 30 40 50 60

60 50 40

B7 TGACTTTGAGCCTTCAGGGTCTGAGGG

::::::::::::: ::::::::::: :

BCR-ABL TGACTTTGAGCCT-CAGGGTCTGAGTG

70 80 90

Translation of the sequence ([3'5' Frame 2](http://web.expasy.org/cgi-bin/translate/dna_sequences?/work/expasy/tmp/http/seqdna.8623,6)):

NDGEGAFHGDAEALQRPVASDFEPSGSE

Alignment of the sequence against BCR-ABL (p190) fusion protein sequence:

**96.2% identity** in 26 aa overlap (1-26:3-28); score: 176 E(10000): 1.7e-13

10 20

B7 NDGEGAFHGDAEALQRPVASDFEPSG

::::::::::::::::::::::::.:

p190 NDGEGAFHGDAEALQRPVASDFEPQG

10 20

**II.8.** # **P377** clone B8

Sequence of the clone (with M13/pUC-R sequencing primer):

CATGCTGATTACGATTCGAGCTCGGTACCCGGGGATCCCTCAGACCCTGAAGGCTCAAAGTCAGATGCTACTGGCCGCTGAAGGGCTTCTGCGTCTCCATGGAAGGCGCCCTCGCCATCGTTGAAGCTTGGCACTGGCCGTCGTTTTACAACGTCGTGACTGGGAAAACCCTGGCGTTACCCAACTTAATCGCCTTGCAGCACATCCCCCTTTCGCCAGCTGGCGTAATAGCGAAGAGGCCCGCACCGATCGCCCTTCCCAACAGTTGCGCAGCCTGAATGGCGAATGGCGCCTGATGCGGTATTTTCTCCTTACGCATCTGTGCGGTATTTCACACCGCATATGGTGCACTCTCAGTACAATCTGCTCTGATGCCGCATAGTTAAGCCAGCCCCGACACCCGCCAACACCCGCTGACGCGCCCTGACGGGCTTGTCTGCTCCCGGCATCCGCTTACAGACAAGCTGTGACCGTCTCCGGGAGCTGCATGTGTCAGAGGTTTTCACCGTCATCACCGAAACGCGCGAGACGAAAGGGCCTCGTGATACGCCTATTTTTATAGGTTAATGTCATGATAATAATGGTTTCTTAGACGTCAGGTGGCACTTTTCGGGGAAATGTGCGCGGAACCCCTATTTGTTTATTTTTCTAAATACATTCAAATATGTATCCGCTCATGAGACAATAACCCTGATAAATGCTTCAATAATATTGAAAAAGGAAGAGTATGAGTATTCAACATTTCCGTGTCGCCCTTATTCCCTTTTTTGCGGCATTTTGCCTTCCTGTTTTTGCTCACCCAGAAACGCTGGTGAAAGTAAAAGATGCTGAAGATCAGTTGGGTGCACGAGTGGGTTACATCGAACTGGATCTCAACAGCGGTAAGATCCTTGAGAGTTTTCGCCCCGAAGAACGTTTTCCAATGATGAGCACTTTTAAAGTTCTGCTATGTGGGCGCGGTATTATCCCGTATTGACGCCCGGGCAAGAGCAACTCGGTCGCCGCATACACTATTTCTCAGAATGACTTTGGTTGAGTACTCACCAGTCACAGAAAAGCATCTTACGGATGGCCATGGACAGTAGAGATTATGCACTGCTTGCATTACATGAGTGAATAACACTGGCGGACCAACCTTACCTTTCTGGAC

Alignment of the sequence against published BCR-ABL (p190) FT sequence:

**97.7% identity** (97.7% similar) in 87 nt overlap (123-37:7-92)

120 110 100 90 80 70

B8 CAACGATGGCGAGGGCGCCTTCCATGGAGACGCAGAAGCCCTTCAGCGGCCAGTAGCATC

::::::::::::::::::::::::::::::::::::::::::::::::::::::::::::

BCR-ABL CAACGATGGCGAGGGCGCCTTCCATGGAGACGCAGAAGCCCTTCAGCGGCCAGTAGCATC

10 20 30 40 50 60

60 50 40

B8 TGACTTTGAGCCTTCAGGGTCTGAGGG

::::::::::::: ::::::::::: :

BCR-ABL TGACTTTGAGCCT-CAGGGTCTGAGTG

70 80 90

Translation of the insert ([3'5' Frame 2](http://web.expasy.org/cgi-bin/translate/dna_sequences?/work/expasy/tmp/http/seqdna.8623,6)):

NDGEGAFHGDAEALQRPVASDFEPSGSE

Alignment of the sequence against BCR-ABL (p190) fusion protein sequence:

**96.2% identity** in 26 aa overlap (1-26:3-28); score: 176 E(10000): 1.7e-13

10 20

B8 NDGEGAFHGDAEALQRPVASDFEPSG

::::::::::::::::::::::::.:

BCR-ABL NDGEGAFHGDAEALQRPVASDFEPQG

10 20

**II.9.** # **P138** clone B1-4

Sequence of the clone (with M13/pUC-R sequencing primer):

CATGAATGATTACGATTCGAGCTCGGTACCCGGGGATCCCTCAGACCCTGAAGGCTCAAAGTCAGATGCTACTGGCCGCTGAAGGGCTTCTGCGTCTCCATGGAAGGCGCCCTCGCCATCGTTGAAGCTTGGCACTGGCCGTCGTTTTACAACGTCGTGACTGGGAAAACCCTGGCGTTACCCAACTTAATCGCCTTGCAGCACATCCCCCTTTCGCCAGCTGGCGTAATAGCGAAGAGGCCCGCACCGATCGCCCTTCCCAACAGTTGCGCAGCCTGAATGGCGAATGGCGCCTGATGCGGTATTTTCTCCTTACGCATCTGTGCGGTATTTCACACCGCATATGGTGCACTCTCAGTACAATCTGCTCTGATGCCGCATAGTTAAGCCAGCCCCGACACCCGCCAACACCCGCTGACGCGCCCTGACGGGCTTGTCTGCTCCCGGCATCCGCTTACAGACAAGCTGTGACCGTCTCCGGGAGCTGCATGTGTCAGAGGTTTTCACCGTCATCACCGAAACGCGCGAGACGAAAGGGCCTCGTGATACGCCTATTTTTATAGGTTAATGTCATGATAATAATGGTTTCTTAGACGTCAGGTGGCACTTTTCGGGGAAATGTGCGCGGAACCCCTATTTGTTTATTTTTCTAAATACATTCAAATATGTATCCGCTCATGAGACAATAACCCTGATAAATGCTTCAATAATATTGAAAAAGGAAGAGTATGAGTATTCAACATTTCCGTGTCGCCCTTATTCCCTTTTTTGCGGCATTTTGCCTTCCTGTTTTTGCTCACCCAGAAACGCTGGTGAAAGTAAAAGATGCTGAAGATCAGTTGGGTGCACGAGTGGGTTACATCGAACTGGATCTCAACAGCGGTAAGATCCTTGAGAGTTTTCGCCCCGAAGAACGTTTTCCAATGATGAGCACTTTTAAAGTTCTGCTATGTGGGCGCGGGTATTATCCCGTATTTGACGCCGGGCAAGAGCAACTCGGTCGCCGCATACACTATTTCTCAGATGACTTGGTTTGAGTACTCACCAGTCACAGAAAAGCATCTTACGGATGCATGACAGTAAGAGAATTATGCATGCTGCATAAGCAATGGAGTGATAACACTGGCCGGACCTAAACCTTAACGT

Alignment of the sequence against published BCR-ABL (p190) FT sequence:

**97.7% identity** (97.7% similar) in 87 nt overlap (124-38:7-92)

120 110 100 90 80 70

B1-4 CAACGATGGCGAGGGCGCCTTCCATGGAGACGCAGAAGCCCTTCAGCGGCCAGTAGCATC

::::::::::::::::::::::::::::::::::::::::::::::::::::::::::::

BCR-ABL CAACGATGGCGAGGGCGCCTTCCATGGAGACGCAGAAGCCCTTCAGCGGCCAGTAGCATC

10 20 30 40 50 60

60 50 40

B1-4 TGACTTTGAGCCTTCAGGGTCTGAGGG

::::::::::::: ::::::::::: :

BCR-ABL TGACTTTGAGCCT-CAGGGTCTGAGTG

70 80 90

Translation of the insert ([3'5' Frame 2](http://web.expasy.org/cgi-bin/translate/dna_sequences?/work/expasy/tmp/http/seqdna.8623,6)):

NDGEGAFHGDAEALQRPVASDFEPSGSE

Alignment of the sequence against BCR-ABL (p190) fusion protein sequence:

**96.2% identity** in 26 aa overlap (1-26:3-28); score: 176 E(10000): 1.7e-13

10 20

B1-4 NDGEGAFHGDAEALQRPVASDFEPSG

::::::::::::::::::::::::.:

p190 NDGEGAFHGDAEALQRPVASDFEPQG

10 20

**II.10.** # **P203** clone B2-1

Sequence of the clone (with M13/pUC-R sequencing primer):

CATGGAATGATTACGATTCGAGCTCGGTACCCGGGGATCCCTCAGACCCTGAAGGCTCAAAGTCAGATGCTACTGGCCGCTGAAGGGCTTCTGCGTCTCCATGGAAGGCGCCCTCGCCATCGTGAAGCTTGGCACTGGCCGTCGTTTTACAACGTCGTGACTGGGAAAACCCTGGCGTTACCCAACTTAATCGCCTTGCAGCACATCCCCCTTTCGCCAGCTGGCGTAATAGCGAAGAGGCCCGCACCGATCGCCCTTCCCAACAGTTGCGCAGCCTGAATGGCGAATGGCGCCTGATGCGGTATTTTCTCCTTACGCATCTGTGCGGTATTTCACACCGCATATGGTGCACTCTCAGTACAATCTGCTCTGATGCCGCATAGTTAAGCCAGCCCCGACACCCGCCAACACCCGCTGACGCGCCCTGACGGGCTTGTCTGCTCCCGGCATCCGCTTACAGACAAGCTGTGACCGTCTCCGGGAGCTGCATGTGTCAGAGGTTTTCACCGTCATCACCGAAACGCGCGAGACGAAAGGGCCTCGTGATACGCCTATTTTTATAGGTTAATGTCATGATAATAATGGTTTCTTAGACGTCAGGTGGCACTTTTCGGGGAAATGTGCGCGGAACCCCTATTTGTTTATTTTTCTAAATACATTCAAATATGTATCCGCTCATGAGACAATAACCCTGATAAATGCTTCAATAATATTGAAAAAGGAAGAGTATGAGTATTCAACATTTCCGTGTCGCCCTTATTCCCTTTTTTGCGGCATTTTGCCTTCCTGTTTTTGCTCACCCAGAAACGCTGGTGAAAGTAAAAGATGCTGAAGATCAGTTGGGTGCACGAGTGGGTTACATCGAACTGGATCTCAACAGCGGTAAGATCCTTGAGAGTTTTCGCCCCGAAGACGTTTTCCAATGATGAGCACTTTTAAAGTTCTGCTATGTGGCGCGGTATTATCCCGGTATTGACGCCGGGCAAGAGCAACTCGGTCGCCGCATACACTATTCTCAGATGACTTGGTTTGAGTACTCACCAGTCACAGAAAAGCATCTTAACGGATGGCCATGACAGTAGAGATAATGCATGCTGGCATATCAATGAGTGAATACACTGGCGGCCAAACTAC

Alignment of the sequence against published BCR-ABL (p190) FT sequence:

**97.6% identity** (97.6% similar) in 85 nt overlap (123-39:9-92)

120 110 100 90 80 70

B2-1 ACGATGGCGAGGGCGCCTTCCATGGAGACGCAGAAGCCCTTCAGCGGCCAGTAGCATCTG

::::::::::::::::::::::::::::::::::::::::::::::::::::::::::::

BCR-ABL ACGATGGCGAGGGCGCCTTCCATGGAGACGCAGAAGCCCTTCAGCGGCCAGTAGCATCTG

10 20 30 40 50 60

60 50 40

B2-1 ACTTTGAGCCTTCAGGGTCTGAGGG

::::::::::: ::::::::::: :

BCR-ABL ACTTTGAGCCT-CAGGGTCTGAGTG

70 80 90

Translation of the insert ([3'5' Frame 1](http://web.expasy.org/cgi-bin/translate/dna_sequences?/work/expasy/tmp/http/seqdna.8623,6)):

HDGEGAFHGDAEALQRPVASDFEPSGSE

Alignment of the sequence against BCR-ABL (p190) fusion protein sequence:

**92.3% identity** in 26 aa overlap (1-26:3-28); score: 170 E(10000): 6.8e-13

10 20

B2-1 HDGEGAFHGDAEALQRPVASDFEPSG

.:::::::::::::::::::::::.:

p190 NDGEGAFHGDAEALQRPVASDFEPQG

10 20

**II.11a.** # **P206** clone B3-1

Sequence of the clone (with M13/pUC-R sequencing primer):

CCCTGCATGATTACGATTCGAGCTCGGTACCCGGGGATCCCTCAGACCCTGAAGGCTCAAAGTCAGATGCTACTGGCCGCTGAAGGGCTTCTGCGTCTCCATGGAAGGCGCCCTCGCCATCGTTGAAGCTTGGCACTGGCCGTCGTTTTACAACGTCGTGACTGGGAAAACCCTGGCGTTACCCAACTTAATCGCCTTGCAGCACATCCCCCTTTCGCCAGCTGGCGTAATAGCGAAGAGGCCCGCACCGATCGCCCTTCCCAACAGTTGCGCAGCCTGAATGGCGAATGGCGCCTGATGCGGTATTTTCTCCTTACGCATCTGTGCGGTATTTCACACCGCATATGGTGCACTCTCAGTACAATCTGCTCTGATGCCGCATAGTTAAGCCAGCCCCGACACCCGCCAACACCCGCTGACGCGCCCTGACGGGCTTGTCTGCTCCCGGCATCCGCTTACAGACAAGCTGTGACCGTCTCCGGGAGCTGCATGTGTCAGAGGTTTTCACCGTCATCACCGAAACGCGCGAGACGAAAGGGCCTCGTGATACGCCTATTTTTATAGGTTAATGTCATGATAATAATGGTTTCTTAGACGTCAGGTGGCACTTTTCGGGGAAATGTGCGCGGAACCCCTATTTGTTTATTTTTCTAAATACATTCAAATATGTATCCGCTCATGAGACAATAACCCTGATAAATGCTTCAATAATATTGAAAAAGGAAGAGTATGAGTATTCAACATTTCCGTGTCGCCCTTATTCCCTTTTTTGCGGCATTTTGCCTTCCTGTTTTTGCTCACCCAGAAACGCTGGTGAAAGTAAAAGATGCTGAAGATCAGTTGGGTGCACGAGTGGGTTACATCGAACTGGATCTCAACAGCGGTAAGATCCTTGAGAGTTTTCGCCCCGAAGAACGTTTTCCAATGATGAGCACTTTTAAAGTTCTGCTATGTGGCGCGGTATTATCCCGTATTGACGCCCGGGCAAGAGCAACTCGGTCGCCGCATACACTATTTCTCAGATGACTTGCTTGAGTACTCCACCAGTTCACAGAAAAAGCAATCTTACGGGATGGGCATGACAGTAAGAAACTATGCCAGTGCCTGGCCATAGCATGAGCTGAATAAACAACTGGCGGGTCA

Alignment of the sequence against published BCR-ABL (p190) FT sequence:

**97.7% identity** (97.7% similar) in 87 nt overlap (125-39:7-92)

120 110 100 90 80 70

B3-1 CAACGATGGCGAGGGCGCCTTCCATGGAGACGCAGAAGCCCTTCAGCGGCCAGTAGCATC

::::::::::::::::::::::::::::::::::::::::::::::::::::::::::::

BCR-ABL CAACGATGGCGAGGGCGCCTTCCATGGAGACGCAGAAGCCCTTCAGCGGCCAGTAGCATC

10 20 30 40 50 60

60 50 40

B3-1 TGACTTTGAGCCTTCAGGGTCTGAGGG

::::::::::::: ::::::::::: :

BCR-ABL TGACTTTGAGCCT-CAGGGTCTGAGTG

70 80 90

Translation of the sequence ([3'5' Frame 2](http://web.expasy.org/cgi-bin/translate/dna_sequences?/work/expasy/tmp/http/seqdna.8623,6)):

NDGEGAFHGDAEALQRPVASDFEPSGSE

Alignment of the sequence against BCR-ABL (p190) fusion protein sequence:

**96.2% identity in 26 aa overlap** (1-26:3-28); score: 176 E(10000): 1.7e-13

10 20

B3-1 NDGEGAFHGDAEALQRPVASDFEPSG

::::::::::::::::::::::::.:

BCR-ABL NDGEGAFHGDAEALQRPVASDFEPQG

10 20

**II.11b.** # **P206** clone B3-2

Sequence of the clone (with M13/pUC-R sequencing primer):

CAAGCCTGATTACGATTCGAGCTCGGTACCCGGGGATCCCTCAGACCCTGAAGGCTCAAAGTCAGATGCTACTGGCCGCTGAAGGGCTTCTGCATCTCCATGGAAGGCGCCCTCGCCATCGTTGAAGCTTGGCACTGGCCGTCGTTTTACAACGTCGTGACTGGGAAAACCCTGGCGTTACCCAACTTAATCGCCTTGCAGCACATCCCCCTTTCGCCAGCTGGCGTAATAGCGAAGAGGCCCGCACCGATCGCCCTTCCCAACAGTTGCGCAGCCTGAATGGCGAATGGCGCCTGATGCGGTATTTTCTCCTTACGCATCTGTGCGGTATTTCACACCGCATATGGTGCACTCTCAGTACAATCTGCTCTGATGCCGCATAGTTAAGCCAGCCCCGACACCCGCCAACACCCGCTGACGCGCCCTGACGGGCTTGTCTGCTCCCGGCATCCGCTTACAGACAAGCTGTGACCGTCTCCGGGAGCTGCATGTGTCAGAGGTTTTCACCGTCATCACCGAAACGCGCGAGACGAAAGGGCCTCGTGATACGCCTATTTTTATAGGTTAATGTCATGATAATAATGGTTTCTTAGACGTCAGGTGGCACTTTTCGGGGAAATGTGCGCGGAACCCCTATTTGTTTATTTTTCTAAATACATTCAAATATGTATCCGCTCATGAGACAATAACCCTGATAAATGCTTCAATAATATTGAAAAAGGAAGAGTATGAGTATTCAACATTTCCGTGTCGCCCTTATTCCCTTTTTTGCGGCATTTTGCCTTCCTGTTTTTGCTCACCCAGAAACGCTGGTGAAAGTAAAAGATGCTGAAGATCAGTTGGGTGCACGAGTGGGTTACATCGAACTGGATCTCAACAGCGGTAAGATCCTTGAGAGTTTTCGCCCCGAAGAACGTTTTCCAATGATGAGCACTTTTAAAGTTCTGCTATGTGGCGCGGTATTATCCCGTATTGACGCCGGGCAAGAGCAACTCGGTCGCCGCATACACTATTTCTCAGATGACTTGGGTTGGAGTACTCACCAGTCACAGAAAAAGCAATCTTACGGATGGGCATGACAGTAAGAGATATGCAGTGCTGGCATACCAATGAGTGATAACCACTGGCGGGACTAT

Alignment of the sequence of the insert against published BCR-ABl (p190) FT sequence:

**97.6% identity** (97.6% similar) in 85 nt overlap (1-85:1-84)

80 70 60 50 40 30

B3-2 CAACGATGGCGAGGGCGCCTTCCATGGAGATGCAGAAGCCCTTCAGCGGCCAGTAGCATC

:::::::::::::::::::::::::::::: :::::::::::::::::::::::::::::

BCR-ABL CAACGATGGCGAGGGCGCCTTCCATGGAGACGCAGAAGCCCTTCAGCGGCCAGTAGCATC

10 20 30 40 50 60

20 10

B3-2 TGACTTTGAGCCTTCAGGGTCTGAG

::::::::::::: :::::::::::

BCR-ABL TGACTTTGAGCCT-CAGGGTCTGAG

70 80

Translation of the insert ([3'5' Frame 2](http://web.expasy.org/cgi-bin/translate/dna_sequences?/work/expasy/tmp/http/seqdna.8623,6)):

NDGEGAFHGDAEALQRPVASDFEPSGSE

Alignment of the sequence against BCR-ABL (p190) fusion protein sequence:

**96.2% identity** in 26 aa overlap (1-26:3-28); score: 176 E(10000): 1.7e-13

10 20

B3-2 NDGEGAFHGDAEALQRPVASDFEPSG

::::::::::::::::::::::::.:

BCR-ABL NDGEGAFHGDAEALQRPVASDFEPQG

10 20

**II.12a.** # **P215** clone B4-2

Sequence of the clone (with M13/pUC-R sequencing primer):

CAGGAATGATTACGATTCGAGCTCGGTACCCGGGGATCCCTCAGACCCTGAAGGCTCAAAGTCAGATGCTACTGGCCGCTGAAGGGCTTCTGCGTCTCCATGGAAGGCGCCCTCGCCATCGTTGAAGCTTGGCACTGGCCGTCGTTTTACAACGTCGTGACTGGGAAAACCCTGGCGTTACCCAACTTAATCGCCTTGCAGCACATCCCCCTTTCGCCAGCTGGCGTAATAGCGAAGAGGCCCGCACCGATCGCCCTTCCCAACAGTTGCGCAGCCTGAATGGCGAATGGCGCCTGATGCGGTATTTTCTCCTTACGCATCTGTGCGGTATTTCACACCGCATATGGTGCACTCTCAGTACAATCTGCTCTGATGCCGCATAGTTAAGCCAGCCCCGACACCCGCCAACACCCGCTGACGCGCCCTGACGGGCTTGTCTGCTCCCGGCATCCGCTTACAGACAAGCTGTGACCGTCTCCGGGAGCTGCATGTGTCAGAGGTTTTCACCGTCATCACCGAAACGCGCGAGACGAAAGGGCCTCGTGATACGCCTATTTTTATAGGTTAATGTCATGATAATAATGGTTTCTTAGACGTCAGGTGGCACTTTTCGGGGAAATGTGCGCGGAACCCCTATTTGTTTATTTTTCTAAATACATTCAAATATGTATCCGCTCATGAGACAATAACCCTGATAAATGCTTCAATAATATTGAAAAAGGAAGAGTATGAGTATTCAACATTTCCGTGTCGCCCTTATTCCCTTTTTTGCGGCATTTTGCCTTCCTGTTTTTGCTCACCCAGAAACGCTGGTGAAAGTAAAAGATGCTGAAGATCAGTTGGGTGCACGAGTGGGTTACATCGAACTGGATCTCAACAGCGGTAAGATCCTTGAGAGTTTTCGCCCCGAAGAACGTTTTCCAATGATGAGCACTTTTAAAGTTCTGCTATGTGGCGCGGTATTATCCCGTATTGACGCCCGGGCAAGAGCAACTCGGTCGCCGCATACACTATTTCTCAGATGACTTGGGTTGAGTACTCACCAGTCACAGAAAAGCATCCTAACGGATGGGCATGACAGTAGAGCATATGCCAGTGCTGGCTATTACCAATGAGTGATAAACCATCTGGCGGGTCA

Alignment of the sequence against published BCR-ABL (p190) FT sequence:

**97.7% identity** (97.7% similar) in 87 nt overlap (124-38:7-92)

120 110 100 90 80 70

B4-2 CAACGATGGCGAGGGCGCCTTCCATGGAGACGCAGAAGCCCTTCAGCGGCCAGTAGCATC

::::::::::::::::::::::::::::::::::::::::::::::::::::::::::::

BCR-ABL CAACGATGGCGAGGGCGCCTTCCATGGAGACGCAGAAGCCCTTCAGCGGCCAGTAGCATC

10 20 30 40 50 60

60 50 40

B4-2 TGACTTTGAGCCTTCAGGGTCTGAGGG

::::::::::::: ::::::::::: :

BCR-ABL TGACTTTGAGCCT-CAGGGTCTGAGTG

70 80 90

Translation of the insert ([3'5' Frame 2](http://web.expasy.org/cgi-bin/translate/dna_sequences?/work/expasy/tmp/http/seqdna.8623,6)):

NDGEGAFHGDAEALQRPVASDFEPSGSE

Alignment of the sequence against BCR-ABL (p190) fusion protein sequence:

**96.2% identity in 26 aa overlap** (1-26:3-28); score: 176 E(10000): 1.7e-13

10 20

B4-2 NDGEGAFHGDAEALQRPVASDFEPSG

::::::::::::::::::::::::.:

p190 NDGEGAFHGDAEALQRPVASDFEPQG

10 20

**II.12b.** # **P215** clone B4-3

Sequence of the clone (with M13/pUC-R sequencing primer):

CAATGAATGATTACGATTCGAGCTCGGTACCCGGGGATCCCTCAGACCCTGAAGGCTCAAAGTCAGATGCTACTGGCCGCTGAAGGGCTTCTGCGTCTCCATGGAAGGCGCCCTCGCCATCGTTGAAGCTTGGCACTGGCCGTCGTTTTACAACGTCGTGACTGGGAAAACCCTGGCGTTACCCAACTTAATCGCCTTGCAGCACATCCCCCTTTCGCCAGCTGGCGTAATAGCGAAGAGGCCCGCACCGATCGCCCTTCCCAACAGTTGCGCAGCCTGAATGGCGAATGGCGCCTGATGCGGTATTTTCTCCTTACGCATCTGTGCGGTATTTCACACCGCATATGGTGCACTCTCAGTACAATCTGCTCTGATGCCGCATAGTTAAGCCAGCCCCGACACCCGCCAACACCCGCTGACGCGCCCTGACGGGCTTGTCTGCTCCCGGCATCCGCTTACAGACAAGCTGTGACCGTCTCCGGGAGCTGCATGTGTCAGAGGTTTTCACCGTCATCACCGAAACGCGCGAGACGAAAGGGCCTCGTGATACGCCTATTTTTATAGGTTAATGTCATGATAATAATGGTTTCTTAGACGTCAGGTGGCACTTTTCGGGGAAATGTGCGCGGAACCCCTATTTGTTTATTTTTCTAAATACATTCAAATATGTATCCGCTCATGAGACAATAACCCTGATAAATGCTTCAATAATATTGAAAAAGGAAGAGTATGAGTATTCAACATTTCCGTGTCGCCCTTATTCCCTTTTTTGCGGCATTTTGCCTTCCTGTTTTTGCTCACCCAGAAACGCTGGTGAAAGTAAAAGATGCTGAAGATCAGTTGGGTGCACGAGTGGGTTACATCGAACTGGATCTCAACAGCGGTAAGATCCTTGAGAGTTTTCGCCCCGAAGAACGTTTTCCAATGATGAGCACTTTTAAAGTTCTGCTATGTGGCGCGGTATTATCCCGTATTGACGCCGGGGCAAGGAGCAACTCGGGTCGCCGCATACACTATTTCTCAGATGACTTTGGTTTGAGTACTCACCAGTCACAGGAAAAAGCAATCCTTACGGGATGGCATGACAGTAGAAGATATGCAGTTGCCTGCCCATTACCCATGAGTGGATTAACACCTGGCAG

Alignment of the sequence against published BCR-ABL (p190) FT sequence:

**97.7% identity** (97.7% similar) in 87 nt overlap (125-39:7-92)

120 110 100 90 80 70

B4-3 CAACGATGGCGAGGGCGCCTTCCATGGAGACGCAGAAGCCCTTCAGCGGCCAGTAGCATC

::::::::::::::::::::::::::::::::::::::::::::::::::::::::::::

BCR-ABL CAACGATGGCGAGGGCGCCTTCCATGGAGACGCAGAAGCCCTTCAGCGGCCAGTAGCATC

10 20 30 40 50 60

60 50 40

B4-3 TGACTTTGAGCCTTCAGGGTCTGAGGG

::::::::::::: ::::::::::: :

BCR-ABL TGACTTTGAGCCT-CAGGGTCTGAGTG

70 80 90

Translation of the sequence ([3'5' Frame 2](http://web.expasy.org/cgi-bin/translate/dna_sequences?/work/expasy/tmp/http/seqdna.8623,6)):

NDGEGAFHGDAEALQRPVASDFEPSGSE

Alignment of the sequence against BCR-ABL (p190) fusion protein sequence:

96.2% identity in 26 aa overlap (1-26:3-28); score: 176 E(10000): 1.7e-13

10 20

8 NDGEGAFHGDAEALQRPVASDFEPSG

::::::::::::::::::::::::.:

p190 NDGEGAFHGDAEALQRPVASDFEPQG

10 20

**II.13a.** # **P239** clone B5-1

Sequence of the clone (with M13/pUC-R sequencing primer):

CATTAATGATTACGAATTCGAGCTCGGTACCCGGGGATCCCCAGACCCTGAAGGCTCAAAGTCAGATGCTACTGGCCGCTGAAGGGCTTCTGCGTCTCCATGGAAGGCGCCCTCGCCATCGTTGAAGCTTGGCACTGGCCGTCGTTTTACAACGTCGTGACTGGGAAAACCCTGGCGTTACCCAACTTAATCGCCTTGCAGCACATCCCCCTTTCGCCAGCTGGCGTAATAGCGAAGAGGCCCGCACCGATCGCCCTTCCCAACAGTTGCGCAGCCTGAATGGCGAATGGCGCCTGATGCGGTATTTTCTCCTTACGCATCTGTGCGGTATTTCACACCGCATATGGTGCACTCTCAGTACAATCTGCTCTGATGCCGCATAGTTAAGCCAGCCCCGACACCCGCCAACACCCGCTGACGCGCCCTGACGGGCTTGTCTGCTCCCGGCATCCGCTTACAGACAAGCTGTGACCGTCTCCGGGAGCTGCATGTGTCAGAGGTTTTCACCGTCATCACCGAAACGCGCGAGACGAAAGGGCCTCGTGATACGCCTATTTTTATAGGTTAATGTCATGATAATAATGGTTTCTTAGACGTCAGGTGGCACTTTTCGGGGAAATGTGCGCGGAACCCCTATTTGTTTATTTTTCTAAATACATTCAAATATGTATCCGCTCATGAGACAATAACCCTGATAAATGCTTCAATAATATTGAAAAAGGAAGAGTATGAGTATTCAACATTTCCGTGTCGCCCTTATTCCCTTTTTTGCGGCATTTTGCCTTCCTGTTTTTGCTCACCCAGAAACGCTGGTGAAAGTAAAAGATGCTGAAGATCAGTTGGGTGCACGAGTGGGTTACATCGAACTGGATCTCAACAGCGGTAAGATCCTTGAGAGTTTTCGCCCCGAAGAACGTTTTCCAATGATGAGCACTTTTAAAGTTCTGCTATGTGGCGCGGTATTATCCCGTATTGACGCCGGGCAAGAGCAACTCGGTCGCCCGCATACACTATTCTCAGAATGACTTTGGTTTGAGTACTCACAGGTCACAGAAAAAAGCATCTTACCGGGATGCCATGGACCAGTAAGAAGCATATGGCCAGTTGCCTTGCATTAACCATTGAGGTGATAAACACTTGGCGGACTC

Alignment of the sequence against published BCR-ABL (p190) FT sequence:

**97.6% identity** (97.6% similar) in 85 nt overlap (124-40:7-90)

120 110 100 90 80 70

B5-1 CAACGATGGCGAGGGCGCCTTCCATGGAGACGCAGAAGCCCTTCAGCGGCCAGTAGCATC

::::::::::::::::::::::::::::::::::::::::::::::::::::::::::::

BCR-ABL CAACGATGGCGAGGGCGCCTTCCATGGAGACGCAGAAGCCCTTCAGCGGCCAGTAGCATC

10 20 30 40 50 60

60 50

B5-1 TGACTTTGAGCCTTCAGGGTCTGGG

::::::::::::: ::::::::: :

BCR-ABL TGACTTTGAGCCT-CAGGGTCTGAG

70 80 90

Translation of the sequence ([3'5' Frame 2](http://web.expasy.org/cgi-bin/translate/dna_sequences?/work/expasy/tmp/http/seqdna.8623,6)):

SFNDGEGAFHGDAEALQRPVASDFEPSGSGDPRVPSSNS-SLM

Alignment of the sequence against BCR-ABL (p190) fusion protein sequence:

96.2% identity in 26 aa overlap (3-28:3-28); score: 176 E(10000): 3.8e-13

10 20

B5-1 NDGEGAFHGDAEALQRPVASDFEPSG

::::::::::::::::::::::::.:

p190 NDGEGAFHGDAEALQRPVASDFEPQG

10 20

**II.13b.** # **P239** clone B5-3

Sequence of the clone (with M13/pUC-R sequencing primer):

GGTGCATGATTACGATTCGAGCTCGGTACCCGGGGATCCCTCAGACCCTGAGGGCTCAAAGTCAGATGCTACTGGCCGCTGAAGGGCTTCTGCGTCTCCATGGAAGGCGCCCTCGCCATCGTTGAAGCTTGGCACTGGCCGTCGTTTTACAACGTCGTGACTGGGAAAACCCTGGCGTTACCCAACTTAATCGCCTTGCAGCACATCCCCCTTTCGCCAGCTGGCGTAATAGCGAAGAGGCCCGCACCGATCGCCCTTCCCAACAGTTGCGCAGCCTGAATGGCGAATGGCGCCTGATGCGGTATTTTCTCCTTACGCATCTGTGCGGTATTTCACACCGCATATGGTGCACTCTCAGTACAATCTGCTCTGATGCCGCATAGTTAAGCCAGCCCCGACACCCGCCAACACCCGCTGACGCGCCCTGACGGGCTTGTCTGCTCCCGGCATCCGCTTACAGACAAGCTGTGACCGTCTCCGGGAGCTGCATGTGTCAGAGGTTTTCACCGTCATCACCGAAACGCGCGAGACGAAAGGGCCTCGTGATACGCCTATTTTTATAGGTTAATGTCATGATAATAATGGTTTCTTAGACGTCAGGTGGCACTTTTCGGGGAAATGTGCGCGGAACCCCTATTTGTTTATTTTTCTAAATACATTCAAATATGTATCCGCTCATGAGACAATAACCCTGATAAATGCTTCAATAATATTGAAAAAGGAAGAGTATGAGTATTCAACATTTCCGTGTCGCCCTTATTCCCTTTTTTGCGGCATTTTGCCTTCCTGTTTTTGCTCACCCAGAAACGCTGGTGAAAGTAAAAGATGCTGAAGATCAGTTGGGTGCACGAGTGGGTTACATCGAACTGGATCTCAACAGCGGTAAGATCCTTGAGAGTTTTCGCCCCGAAGAACGTTTTCCAATGATGAGCACTTTTAAAGTTCTGCTATGTGGCGCGGTATTATCCCGTATTGACGCCGGGCAAGAGCAACTCGGTCGCCGCATACACTATTCTCAGAATGACTTGGTTGAGTACTCACCAGTCACAAGAAAAAGCATCCTACCGAATGCCATGACAGTAGAGATATGCATGCTGCATTACAATGAGTGATAACACTGGCGGATCCTAT

Alignment of the sequence against published BCR-ABL (p190) FT sequence:

**97.7% identity** (97.7% similar) in 87 nt overlap (124-38:7-92)

120 110 100 90 80 70

B5-3 CAACGATGGCGAGGGCGCCTTCCATGGAGACGCAGAAGCCCTTCAGCGGCCAGTAGCATC

::::::::::::::::::::::::::::::::::::::::::::::::::::::::::::

BCR-ABL CAACGATGGCGAGGGCGCCTTCCATGGAGACGCAGAAGCCCTTCAGCGGCCAGTAGCATC

10 20 30 40 50 60

60 50 40

B5-3 TGACTTTGAGCCCTCAGGGTCTGAGGG

:::::::::::: :::::::::::: :

BCR-ABL TGACTTTGAGCC-TCAGGGTCTGAGTG

70 80 90

Translation of the insert ([3'5' Frame 2](http://web.expasy.org/cgi-bin/translate/dna_sequences?/work/expasy/tmp/http/seqdna.8623,6)):

NDGEGAFHGDAEALQRPVASDFEPSGSE

Alignment of the sequence against BCR-ABL (p190) fusion protein sequence:

96.2% identity in 26 aa overlap (1-26:3-28); score: 176 E(10000): 1.7e-13

10 20

B5-3 NDGEGAFHGDAEALQRPVASDFEPSG

::::::::::::::::::::::::.:

p190 NDGEGAFHGDAEALQRPVASDFEPQG

10 20

**II.14a.** # **P310** clone B6-1

Sequence of the clone (with M13/pUC-R sequencing primer):

GAGTTATGATTACGATTCGAGCTCGGTACCCGGGGATCCCTCAGACCCTGAAGGCTCAAAGTCAGATGCTACTGGCCGCTGAAGGGCTTCTGCGTCTCCATGGAAGGCGCCCTCGCCATCGTTGAAGCTTGGCACTGGCCGTCGTTTTACAACGTCGTGACTGGGAAAACCCTGGCGTTACCCAACTTAATCGCCTTGCAGCACATCCCCCTTTCGCCAGCTGGCGTAATAGCGAAGAGGCCCGCACCGATCGCCCTTCCCAACAGTTGCGCAGCCTGAATGGCGAATGGCGCCTGATGCGGTATTTTCTCCTTACGCATCTGTGCGGTATTTCACACCGCATATGGTGCACTCTCAGTACAATCTGCTCTGATGCCGCATAGTTAAGCCAGCCCCGACACCCGCCAACACCCGCTGACGCGCCCTGACGGGCTTGTCTGCTCCCGGCATCCGCTTACAGACAAGCTGTGACCGTCTCCGGGAGCTGCATGTGTCAGAGGTTTTCACCGTCATCACCGAAACGCGCGAGACGAAAGGGCCTCGTGATACGCCTATTTTTATAGGTTAATGTCATGATAATAATGGTTTCTTAGACGTCAGGTGGCACTTTTCGGGGAAATGTGCGCGGAACCCCTATTTGTTTATTTTTCTAAATACATTCAAATATGTATCCGCTCATGAGACAATAACCCTGATAAATGCTTCAATAATATTGAAAAAGGAAGAGTATGAGTATTCAACATTTCCGTGTCGCCCTTATTCCCTTTTTTGCGGCATTTTGCCTTCCTGTTTTTGCTCACCCAGAAACGCTGGTGAAAGTAAAAGATGCTGAAGATCAGTTGGGTGCACGAGTGGGTTACATCGAACTGGATCTCAACAGCGGTAAGATCCTTGAGAGTTTTCGCCCCGAAGAACGTTTTCCAATGATGAGCACTTTTAAGTTCTGCTATGTGGCGCGTATTTATCCCGTATTGACGCCGGGCAAGAGCAACTCGGTCGCCGCATACACTATTTCTCAGATGACTTTGGTTGAGTACTTCACCAGTCACAGAAAAAGCCATCCTACCGGAATGGGCATGACAGTAGACATATGGCAGTGCCTGCATTAGCCAATGGAGGTGATTAACCATCTGGCCGGG

Alignment of the sequence against published BCR-ABL (p190) FT sequence:

**97.7% identity** (97.7% similar) in 87 nt overlap (124-38:7-92)

120 110 100 90 80 70

B6-1 CAACGATGGCGAGGGCGCCTTCCATGGAGACGCAGAAGCCCTTCAGCGGCCAGTAGCATC

::::::::::::::::::::::::::::::::::::::::::::::::::::::::::::

BCR-ABL CAACGATGGCGAGGGCGCCTTCCATGGAGACGCAGAAGCCCTTCAGCGGCCAGTAGCATC

10 20 30 40 50 60

60 50 40

B6-1 TGACTTTGAGCCCTCAGGGTCTGAGGG

:::::::::::: :::::::::::: :

BCR-ABL TGACTTTGAGCC-TCAGGGTCTGAGTG

Translation of the insert ([3'5' Frame 2](http://web.expasy.org/cgi-bin/translate/dna_sequences?/work/expasy/tmp/http/seqdna.8623,6)):

NDGEGAFHGDAEALQRPVASDFEPSGSE

Alignment of the sequence against BCR-ABL (p190) fusion protein sequence:

**96.2% identity** in 26 aa overlap (1-26:3-28); score: 176 E(10000): 1.7e-13

10 20

B6-1 NDGEGAFHGDAEALQRPVASDFEPSG

::::::::::::::::::::::::.:

p190 NDGEGAFHGDAEALQRPVASDFEPQG

10 20

**II.14a.** # **P310** clone B6-2

Sequence of the clone (with M13/pUC-R sequencing primer):

CATGCTGATTACGATTCGAGCTCGGTACCCGGGGATCCCTCAGACCCTGAAGGCTCAAAGTCAGATGCTACTGGCCGCTGAAGGGCTTCTGCGTCTCCATGGAAGGCGCCCTCGCCATCGTTGAAGCTTGGCACTGGCCGTCGTTTTACAACGTCGTGACTGGGAAAACCCTGGCGTTACCCAACTTAATCGCCTTGCAGCACATCCCCCTTTCGCCAGCTGGCGTAATAGCGAAGAGGCCCGCACCGATCGCCCTTCCCAACAGTTGCGCAGCCTGAATGGCGAATGGCGCCTGATGCGGTATTTTCTCCTTACGCATCTGTGCGGTATTTCACACCGCATATGGTGCACTCTCAGTACAATCTGCTCTGATGCCGCATAGTTAAGCCAGCCCCGACACCCGCCAACACCCGCTGACGCGCCCTGACGGGCTTGTCTGCTCCCGGCATCCGCTTACAGACAAGCTGTGACCGTCTCCGGGAGCTGCATGTGTCAGAGGTTTTCACCGTCATCACCGAAACGCGCGAGACGAAAGGGCCTCGTGATACGCCTATTTTTATAGGTTAATGTCATGATAATAATGGTTTCTTAGACGTCAGGTGGCACTTTTCGGGGAAATGTGCGCGGAACCCCTATTTGTTTATTTTTCTAAATACATTCAAATATGTATCCGCTCATGAGACAATAACCCTGATAAATGCTTCAATAATATTGAAAAAGGAAGAGTATGAGTATTCAACATTTCCGTGTCGCCCTTATTCCCTTTTTTGCGGCATTTTGCCTTCCTGTTTTTGCTCACCCAGAAACGCTGGTGAAAGTAAAAGATGCTGAAGATCAGTTGGGTGCACGAGTGGGTTACATCGAACTGGATCTCAACAGCGGTAAGATCCTTGAGAGTTTTCGCCCCGAAGAACGTTTTCCAATGATGAGCACTTTTAAAGTTCTGCTATGTGGCGCGGTATTATCCCGTATTGACGCCCGGGCAAGAGCAACTCGGTCGCCGCATACACTATTCTCAGAATGACTTTGGCTTGAGTACTCACAGTCACAGAAAAAGCCATCCTACCGGATGCATGACAGTAGAGATATGCAGTGCTGGCATAGCAATGAGTGAATAACACTGGGCGGATCA

Alignment of the sequence against published BCR-ABL (p190) FT sequence:

**97.7% identity** (97.7% similar) in 87 nt overlap (123-37:7-92)

120 110 100 90 80 70

B6-2 CAACGATGGCGAGGGCGCCTTCCATGGAGACGCAGAAGCCCTTCAGCGGCCAGTAGCATC

::::::::::::::::::::::::::::::::::::::::::::::::::::::::::::

BCR-ABL CAACGATGGCGAGGGCGCCTTCCATGGAGACGCAGAAGCCCTTCAGCGGCCAGTAGCATC

10 20 30 40 50 60

60 50 40

B6-2 TGACTTTGAGCCTTCAGGGTCTGAGGG

::::::::::::: ::::::::::: :

BCR-ABL TGACTTTGAGCCT-CAGGGTCTGAGTG

70 80 90

Translation of the sequence ([3'5' Frame 2](http://web.expasy.org/cgi-bin/translate/dna_sequences?/work/expasy/tmp/http/seqdna.8623,6)):

NDGEGAFHGDAEALQRPVASDFEPSGSE

Alignment of the sequence against BCR-ABL (p190) fusion protein sequence:

**96.2% identity** in 26 aa overlap (326-351:3-28); score: 176 E(10000): 1.8e-12

330 340 350

B6-2 NDGEGAFHGDAEALQRPVASDFEPSG

::::::::::::::::::::::::.:

p190 NDGEGAFHGDAEALQRPVASDFEPQG

1. 20

SUMMARY of BCR-ABL (p190) sequencing:

It is obvious (from the sequencing profiles) that extra T is present in all sequencings of the BCR-ABL (p190) clones . This extra T comes from the incorrect reverse primer ABL-Rseq which was used to amplify the R-T qPCR product, its sequence is as follows:

ABL-Rseq: ATG GAT CCC TCA GAC CCT G(A)A GGC TCA A  (incorrect)

ABL-Rseq: ATG GAT CCC TCA GAC CCT GA GGC TCA A (correct)

BCR-Fseq: TTA AGC TTC AAC GAT GGC GAG GGC G

It means that extra A present in ABL-Rseq primer was transferred into PCR product during amplification of R-T qPCR product, these two primers, i.e BCR-Fseq and ABL-Rseq were used for standard PCR amplification of qPCR product with introduction of RE-sites (underlined) into pUC18 vector (GGATCC = BamHI, AAGCTT = HindIII)

...........................................................................

1. **MLL-AF4:** P146

**III.1a.** # **P146** clone M1(1)

Sequence of the clone (with M13/pUC-R sequencing primer):

GATCGGGCCAGTGCAGCTTGGAGTCCACAGGATCAGAGTGGACTTTAAGCAGACCTACTCCAATGAAGTCCATTGTGTTGAAAAGATTCTGAAGGAAATGACCCATTCATGGCCGCCTCCTTTGACAGCAATACATACGCCTAGTACAGCTGAGCCATCCAAGTTTCCGAATTCGTAATCATGTCATAGCTGTTTCCTGTGTGAAATTGTTATCCGCTCACAATTCCACACAACATACGAGCCGGAAGCATAAAGTGTAAAGCCTGGGGTGCCTAATGAGTGAGCTAACTCACATTAATTGCGTTGCGCTCACTGCCCGCTTTCCAGTCGGGAAACCTGTCGGGCCAGCTGCATTAATGAATCGGCCAACGCGCGGGGAGAGGCGGTTTGCGTATTGGGCGCTCTTCCGCTTCCTCGCTCACTGACTCGCTGCGCTCGGTCGTTCGGCTGCGGCGAGCGGTATCAGCTCACTCAAAGGCGGTAATACGGTTATCCACAGAATCAGGGGATAACGCAGGAAAGAACATGTGAGCAAAAGGCCAGCAAAAGGCCAGGAACCGTAAAAAGGCCGCGTTGCTGGCGTTTTTCCATAGGCTCCGCCCCCCTGACGAGCATCACAAAAATCGACGCTCAAGTCAGAGGTGGCGAAACCCGACAGGACTATAAAGATACCAGGCGTTTCCCCCTGGAAGCTCCCTCGTGCGCTCTCCTGTTCCGACCCTGCCGCTTACCGGATACCTGTCCGCCTTTCTCCCTTCGGGAAGCGTGGCGCTTTCTCATAGCTCACGCTGTAGGTATCTCAGTTCGGTGTAGGTCGTTCGCTCCAAGCTGGGCTGTGTGCACGAACCCCCCGTTCAGCCCGACCGCTGCGCCCTTATCCGGGTAACTATCGTCTTGAGTCCAACCCGGGTAAGACACGACTTATCGCCACTGGCAGCAGCCACTGATAACAGGATTAGCAGAGCGGAGTATGTAGGCGGTGCTACAGAGTTCTTGAAGTGGTGCCCTTACTACCGCCTACACTAGAGACAGTATTTGATATCTGCGCCTCTGCTGAAGCAAGTAACTCCGAAGGATTGCTAGCTCTTGACTCCGTCAAACAATCCACTCGCCTTGGCATAAGC

Alignment of the sequence against published MLL-AF4 FT sequence:

**99.3% identity** (99.3% similar) in 150 nt overlap (19-168:3-152)

20 30 40 50 60 70

M1(1) TGGAGTCCACAGGATCAGAGTGGACTTTAAGCAGACCTACTCCAATGAAGTCCATTGTGT

::::::::::::::::::::::::::::::::::::::::::::::::::::::::::::

MLL-AF4 TGGAGTCCACAGGATCAGAGTGGACTTTAAGCAGACCTACTCCAATGAAGTCCATTGTGT

10 20 30 40 50 60

80 90 100 110 120 130

M1(1) TGAAAAGATTCTGAAGGAAATGACCCATTCATGGCCGCCTCCTTTGACAGCAATACATAC

:::: :::::::::::::::::::::::::::::::::::::::::::::::::::::::

MLL-AF4 TGAAGAGATTCTGAAGGAAATGACCCATTCATGGCCGCCTCCTTTGACAGCAATACATAC

70 80 90 100 110 120

140 150 160

M1(1) GCCTAGTACAGCTGAGCCATCCAAGTTTCC

::::::::::::::::::::::::::::::

MLL-AF4 GCCTAGTACAGCTGAGCCATCCAAGTTTCC

130 140 150

Translation of the sequence ([3'5' Frame 2](http://web.expasy.org/cgi-bin/translate/dna_sequences?/work/expasy/tmp/http/seqdna.8623,6)):

IGPVQLGVHRIRVDFKQTYSNEVHCVEKILKEMTHSWPPPLTAIHTPSTAEPSKFPNS-S

CHSCFLCEIVIRSQFHTTYEPEA-SVKPGVPNE-ANSH-LRCAHCPLSSRETCRASCINE

SANARGEAVCVLGALPLPRSLTRCARSFGCGERYQLTQRR-YGYPQNQGITQERTCEQKA

SKRPGTVKRPRCWRFSIGSAPLTSITKIDAQVRGGETRQDYKDTRRFPLEAPSCALLFRP

CRLPDTCPPFSLREAWRFLIAHAVGISVRCRSFAPSWAVCTNPPFSPTAAPLSG-LSS-V

QPG-DTTYRHWQQPLITGLAERSM-AVLQSS-SGALTTAYTRDSI-YLRLC-SK-LRRIA

SS-LRQTIHSPWHK

Alignment of the sequence against MLL-AF4 fusion protein sequence:

**98.0% identity in 50 aa overlap** (7-56:2-51); score: 346 E(10000): 1.2e-28

10 20 30 40 50

M1(1) GVHRIRVDFKQTYSNEVHCVEKILKEMTHSWPPPLTAIHTPSTAEPSKFP

:::::::::::::::::::::.::::::::::::::::::::::::::::

MLL-AF GVHRIRVDFKQTYSNEVHCVEEILKEMTHSWPPPLTAIHTPSTAEPSKFP

10 20 30 40 50

**III.1b.** # **P146** clone M1(2)

Sequence of the clone (with M13/pUC-F sequencing primer):

CAGCATGATTACGATTCGGAACTTGGATGGCTCAGCTGTACTAGGCGTATGTATTGCTGTCAAAGGAGGCGGCCATGAATGGGTCATTTCCTTCAGAATCTCTTCAACACAATGGACTTCATTGGAGTAGGTCTGCTTAAAGTCCACTCTGATCCTGTGGACTCCAAGCTTGGCACTGGCCGTCGTTTTACAACGTCGTGACTGGGAAAACCCTGGCGTTACCCAACTTAATCGCCTTGCAGCACATCCCCCTTTCGCCAGCTGGCGTAATAGCGAAGAGGCCCGCACCGATCGCCCTTCCCAACAGTTGCGCAGCCTGAATGGCGAATGGCGCCTGATGCGGTATTTTCTCCTTACGCATCTGTGCGGTATTTCACACCGCATATGGTGCACTCTCAGTACAATCTGCTCTGATGCCGCATAGTTAAGCCAGCCCCGACACCCGCCAACACCCGCTGACGCGCCCTGACGGGCTTGTCTGCTCCCGGCATCCGCTTACAGACAAGCTGTGACCGTCTCCGGGAGCTGCATGTGTCAGAGGTTTTCACCGTCATCACCGAAACGCGCGAGACGAAAGGGCCTCGTGATACGCCTATTTTTATAGGTTAATGTCATGATAATAATGGTTTCTTAGACGTCAGGTGGCACTTTTCGGGGAAATGTGCGCGGAACCCCTATTTGTTTATTTTTCTAAATACATTCAAATATGTATCCGCTCATGAGACAATAACCCTGATAAATGCTTCAATAATATTGAAAAAGGAAGAGTATGAGTATTCAACATTTCCGTGTCGCCCTTATTCCCTTTTTTGCGGCATTTTGCCTTCCTGTTTTTGCTCACCCAGAAACGCTGGTGAAAGTAAAAGATGCTGAAGATCAGTTGGGTGCACGAGTGGTTTACATCGAAACTGGATCTCAACAGCGGTAAGATCCTTTGAGAGTTTTCGCCCCGAAGACGGTTTTCCAATGATGAGCACTTTTAAAGGTTCTGCCTATGTTGCGCGTATATCCCGTATGAACGCGGCCAGAGCCACTCGGTCGCCGCATACACTATTCTCAGATGGACTGTGAGTACTCACAGTCACAGAAAGCATTCTTACGGGGATG

Alignment of the sequence against published MLL-AF4 FT sequence:

**99.3% identity** (99.3% similar) in 149 nt overlap (166-18:3-151)

160 150 140 130 120 110

M1(2) TGGAGTCCACAGGATCAGAGTGGACTTTAAGCAGACCTACTCCAATGAAGTCCATTGTGT

::::::::::::::::::::::::::::::::::::::::::::::::::::::::::::

MLL-AF4 TGGAGTCCACAGGATCAGAGTGGACTTTAAGCAGACCTACTCCAATGAAGTCCATTGTGT

10 20 30 40 50 60

100 90 80 70 60 50

M1(2) TGAAGAGATTCTGAAGGAAATGACCCATTCATGGCCGCCTCCTTTGACAGCAATACATAC

::::::::::::::::::::::::::::::::::::::::::::::::::::::::::::

MLL-AF4 TGAAGAGATTCTGAAGGAAATGACCCATTCATGGCCGCCTCCTTTGACAGCAATACATAC

70 80 90 100 110 120

40 30 20

M1(2) GCCTAGTACAGCTGAGCCATCCAAGTTCC

::::::::::::::::::::::::::: :

MLL-AF4 GCCTAGTACAGCTGAGCCATCCAAGTTTC

130 140 150

Sequence from START to BamHI-cloning site:

CAGCATGATTACGATTCGGAACTTGGATGGCTCAGCTGTACTAGGCGTATGTATTGCTGTCAAAGGAGGCGGCCATGAATGGGTCATTTCCTTCAGAATCTCTTCAACACAATGGACTTCATTGGAGTAGGTCTGCTTAAAGTCCACTCTGATCCTGTGGACTCC

Translation of the sequence ([3'5' Frame 1](http://web.expasy.org/cgi-bin/translate/dna_sequences?/work/expasy/tmp/http/seqdna.8623,6)):

GVHRIRVDFKQTYSNEVHCVEEILKEMTHSWPPPLTAIHTPSTAEPSKFRIVIML

Alignment of the sequence against MLL-AF4 fusion protein sequence:

**100.0% identity in 49 aa overlap (1-49:2-50); score: 341 E(10000): 4.4e-29**

10 20 30 40

M1(2) GVHRIRVDFKQTYSNEVHCVEEILKEMTHSWPPPLTAIHTPSTAEPSKF

:::::::::::::::::::::::::::::::::::::::::::::::::

MLL-AF4 GVHRIRVDFKQTYSNEVHCVEEILKEMTHSWPPPLTAIHTPSTAEPSKF

10 20 30 40 50
